# Lipidomic Signatures Reveal Seasonal Shifts on the Relative Abundance of High-Valued Lipids from the Brown Algae *Fucus vesiculosus*

**DOI:** 10.3390/md17060335

**Published:** 2019-06-04

**Authors:** Elisabete da Costa, Pedro Domingues, Tânia Melo, Elisabete Coelho, Rui Pereira, Ricardo Calado, Maria H. Abreu, M. Rosário Domingues

**Affiliations:** 1 Centro de Espetrometria de Massa, Departamento de Química & QOPNA& LAQV-REQUIMTE, Universidade de Aveiro, Campus Universitário de Santiago, 3810-193 Aveiro, Portugal; elisabetecosta@ua.pt (E.d.C.) ; p.domingues@ua.pt (P.D.); taniamelo@ua.pt (T.M.); 2 Departamento de Química & CESAM & ECOMARE, Universidade de Aveiro, Campus Universitário de Santiago, 3810-193 Aveiro, Portugal; 3 Departamento de Química & QOPNA & LAQV-REQUIMTE, Universidade de Aveiro, Campus Universitário de Santiago, 3810-193 Aveiro, Portugal; ecoelho@ua.pt; 4 ALGAplus-Produção e comercialização de algas e seus derivados, Lda., 3830-196 Ílhavo, Portugal; rgpereira@algaplus.pt (R.P.); htabreu@algaplus.pt (M.H.A.); 5 Departamento de Biologia & CESAM & ECOMARE, Universidade de Aveiro, Campus Universitário de Santiago, 3810-193 Aveiro, Portugal; rjcalado@ua.pt

**Keywords:** acclimatisation, phospholipids, glycolipids, betaine lipids, season, active ingredients

## Abstract

*Fucus vesiculosus* is an edible brown macroalga, with health benefits associated with its consumption and also a source of bioactive molecules. It is acknowledged that the biochemical composition of macroalgae changes when exposed to different environmental conditions occurring on different habitats, such as the water temperature, and light intensity. In the present study, the polar lipidome of *Fucus vesiculosus* was characterized for the first time using modern high-resolution HILIC–MS, and MS/MS approaches, to evaluate the phenotypic variability in two seasons of the year, e.g., winter and spring. A total of 187 molecular species were identified over eighteen classes of glycolipids, phospholipids and betaine lipids. Principal component analysis (PCA) multivariate statistical analysis and cluster analysis of polar lipid classes, polar lipid species and total fatty acids (FA) datasets, showed clustering according to the seasonal groups. While the lipid profile of *Fucus vesiculosus* harvested in the winter and spring yielded the same molecular species, the relative abundance of these species was significantly different. In the winter, changes were mainly due to the increased relative abundance of some molecular species of glycolipids and phospholipids, bearing octadeca(poly)enoic (18:3, 18:4) and eicosa(poly)enoic (20:4, 20:5) FA and betaine lipids species with short saturated FA (14:0) and polyunsaturated FA (PUFA). Importantly, glycolipids with *n*-3 PUFA and sulfolipids, have been reported to have important biological activities and therapeutic value. Overall, *Fucus vesiculosus* is a promising source of bioactive compounds that can be used as functional food or ingredients for human nutrition, feed, pharma, and cosmetic formulations. In this study, samples harvested in the winter season maximized yields of these bioactive components, when compared with samples harvested in the spring.

## 1. Introduction

The *Fucus vesiculosus* Linnaeus (1753), popularly known as bladderwrack, is an edible brown macroalga (Ochrophyta, Phaeophyceae) that is commonly present on the middle shores of the western Baltic Sea, Atlantic coasts of North America, Europe, and Western Mediterranean [1,2]. This macroalga has been used for food and feed in Europe since the 17th century, as well as in traditional medicine in China [3]. It is currently used as a raw material for various industries, targeting food processing, the development of new pharmaceuticals and cosmetics, along with other new products to high-end markets [4,5,6,7]. The culture of this macroalga is attracting a growing interest in Europe, mainly due to its effect on reducing the environmental impact of intensive agriculture and fish aquaculture and eutrophication [8,9,10]. The production of *Fucus vesiculosus* using land-based integrated multi-trophic aquaculture (IMTA) is considered a sustainable alternative to producing this macroalga [11]. 

*Fucus vesiculosus* has already been reported to be an important source of iodine [12], polysaccharides, e.g., fucoidans [13,14] and alginic acid, phlorotannins [1], and also high-valued molecules such as lipids (1–5% per dry biomass) [15,16,17,18]. The lipid fraction of *F. vesiculosus* is characterized by a high amount of essential polyunsaturated fatty acids (PUFA), such as linoleic (18:2*n*-6), α-linolenic (18:3*n*-3) and octadecatetraenoic (18:4*n*-3) acids, as well arachidonic (20:4*n*-6) and eicosapentaenoic (20:5*n*-3) fatty acids, which grant this brown macroalgae high nutritional value [15,16,17]. Polyunsaturated fatty acids are important in the prevention of several diseases, such as cardiovascular diseases and cancer [19,20], and the consumption of this brown macroalgae is often identified as a healthier choice option.

In macroalgae, fatty acids are mostly esterified to more complex lipids such as glycoglycerolipids (GLs), glycerophospholipids (PLs), and betaine lipids. These polar lipids are structural components of cell membranes and organelles, namely chloroplasts [21], and act as sources of PUFA but also have important health benefits. Polar lipids from brown macroalgae have been reported to have anti-inflammatory and antitumoral activities [4,22,23,24,25]. More recently, some polar lipid classes such as betaine lipids, phosphatidylethanolamine, and phosphatidylcholine phospholipids were identified in the organic solvent extracts of *F. vesiculosus*, that showed an antimicrobial effect against the ESKAPE panel of bacterial, fungal and algal pathogens [26]. These organic solvent extracts also showed a significant inhibitory effect on the pancreatic cancer cell line Panc1, with higher induction effect observed in extracts of samples collected in the winter and end of spring seasons [26]. The knowledge on the polar lipid composition of *Fucus* is an essential step for the discovery of lipidic bioactive compounds and new bioactive properties, fostering the bioprospection of lipidic extracts.

The lipid metabolism of macroalgae is modulated in response to changes in environmental factors such as temperature and light [27,28,29,30,31,32,33,34]. The variation of lipids with season was evaluated in the brown alga *Fucus serratus*. It was reported that low light irradiance and a shorter light period can induce an accumulation of glycolipids monogalactosyldiacylglycerol (MGDG), digalactosyldiacylglycerol (DGDG) and sulfoquinovosyldiacylglycerol (SQDG), and phospholipids phosphatidylglycerol (PG), phosphatidylcholine (PC) and phosphatidylethanolamine (PE), both present in photosynthetic membranes [33]. Concerning the effect of temperature, it was reported for *Fucus* sp. that cold water temperature can induce an increase of degree of fatty acid unsaturation [33,35,36].

So far, the lipidome from *F. vesiculosus* and the seasonal effect on its profile was mainly studied using the FA composition in total lipid extracts, and after separation of lipid class by TLC [16] but never has it been addressed at the molecular level of each molecular species. The identification of the lipid composition at the molecular level can be effectively achieved by using a liquid chromatography-mass spectrometry (LC–MS)-based lipidomic approach. This approach has been successfully used to characterize the lipidome profile of several macroalgae such as *Chondrus crispus* (Florideophyceae) [37], *Codium tomentosum* (Bryopsidophyceae) [38], *Gracilaria* sp. (Florideophyceae) [39], *Porphyra dioica* (Bangiophyceae) [40], *Ulva* sp. (Ulvophyceae) [41], fostering the valorization and the identification of potential valuable lipids in macroalgae.

In this study, we identified the lipidome of *F. vesiculosus* by using hydrophilic interaction chromatography (HILIC)–LC coupled with high-resolution mass spectrometry (HRMS) and evaluated the effects of seasonal variation, e.g., to winter versus spring seasons, on the lipidome. *Fucus vesiculosus* was collected on a land-based integrated aquaculture framework at two different sampling occasions: February (winter) and May (spring) collections. The result gathered in the present work will contribute to enhancing the knowledge on the nutritional value of this alga as food and for the bioprospection of target active ingredients for application in pharmaceutical, cosmetical and cosmeceutical industry.

## 2. Results

### 2.1. Recovery of Total Lipids

The total lipid extracts obtained from *F. vesiculosus* collected in February (winter), and May (spring) accounted for 1.74 ± 0.25% and 1.47 ± 0.17% of the dry weight biomass (DW), respectively. These values were concordant with the lipid content range previously reported for *Fucus* species (0.4 up to 4% DW) [16,33]. A significant difference was observed in the total lipid extract recovery depending on the season (*, p < 0.05), with a higher yield observed in the winter season.

### 2.2. Identification of Polar Lipids Profile from Fucus vesiculosus

The analysis of the total lipid extracts from *F. vesiculosus* by LC-MS, and MS/MS allowed identifying 187 molecular species distributed by eighteen classes, including six classes of glycolipids (GL), eight classes of phospholipids (PL), and four classes of betaine lipids (Supplementary information, Table S1). The criteria for identifying the lipid species included the accuracy of the mass measurements (< 5 ppm), the LC retention time (Supplementary information, Figures S1, S2, S6, S7, S10) and the characteristics of the MS/MS spectra. The manual analysis of the MS/MS spectra allowed the confirmation of the polar head and fatty acyl composition, as previously reported [37,38,41]. Overall, the same classes and lipid species were identified in the lipidome of *F. vesiculosus* collected in winter and in spring, but with significant differences of the relative abundance of same lipid species, as will be described below.

**Figure 1:**
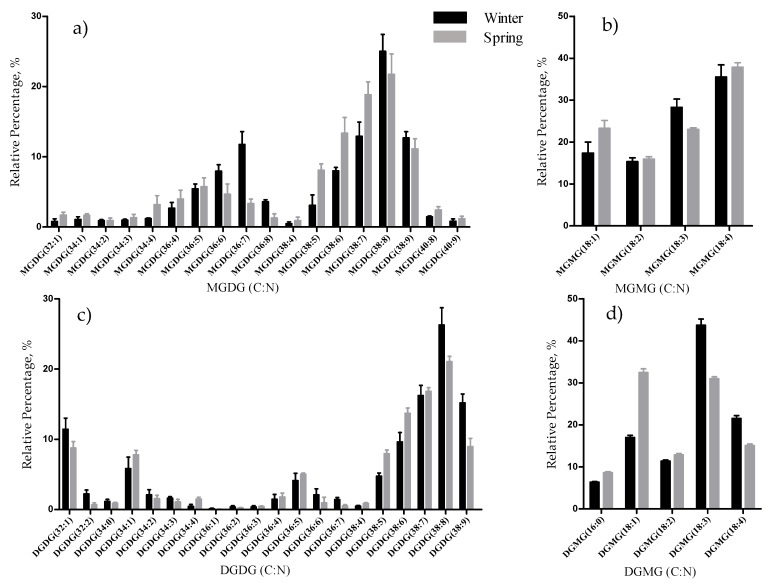
Relative abundance (RA) of molecular species of glycolipids identified in *Fucus vesiculosus* collected in winter (February) and spring (May), determined by HILIC LC–MS: (**a**) MGDG, (**b**) MGMG plus (**c**) DGMG, and (**d**) DGDG molecular species. Numbers in parentheses (C:N) indicates the number of carbon atoms (C) and double bonds (N) in the fatty acid side chains. Species with higher RA were highly unsaturated species: MGDG (38:6) to (38:9) (**a**), and DGDG (38:6) to (38:9) (**c**). The lyso-galactolipids species with higher relative abundance (RA) were MGMG (18:4) (**b**) and DGMG (18:1) and (18:3) (**d**).

The glycolipids (GL) identified in the lipidome of *F. vesiculosus* included both neutral galactolipids and acidic glycolipids. The neutral GL (62 species), identified as [M + NH_4_]^+^ ions in the MS spectra (Supplementary information, Figures S3 and S4), were assigned as monogalactosyldiacylglycerol (MGDG, 26 species), lyso-MGDG (MGMG, 4 species), digalactosyldiacylglycerol (DGDG, 27), and lyso-DGDG (DGMG, 5 species). The neutral GL included molecular species with FA chain lengths ranging from C14 to C20 and the degree of unsaturations from 0 to 5. The most abundant species per class were the MGDG (38:8) at *m/z* 816.6, the DGDG (38:8) at *m/z* 978.6, the MGMG (18:4) observed at *m/z* 530.3 and DGMG (18:3) at *m/z* 694.4 (winter season) and DGMG (18:1) (spring season), (Table 1; Figure 1). Notably, the most abundant MGDG and DGDG species contained highly unsaturated FA.

**Figure 2:**
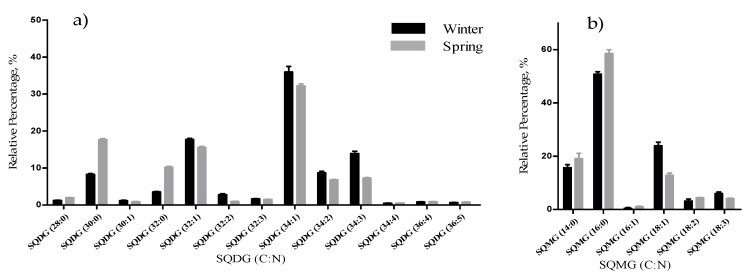
Relative abundance (RA) of molecular species of sulfolipids identified in *F. vesiculosus* collected in winter (February) and spring (May), determined by HILIC LC-MS: (**a**) SQDG; (**b**) SQMG). Numbers in parentheses (C:N) indicates the number of carbon atoms (C) and double bonds (N) in the fatty acid side chains. The species with higher relative abundance were SQDG (34:1) (**a**) and SQMG (16:0) (**b**).

The acidic GL sulfolipids (19 species) were identified as [M − H]^−^ ions in the MS spectra and assigned to sulfoquinovosyldiacylglycerol (SQDG, 13 species) and lyso-SQDG (SQMG, 6 species) (Table 1, Supplementary information Figures S3 and S5). The most abundant species were SQDG (34:1) assigned as SQDG 18:1/16:0 (*m/z* 819.5) and the SQMG (16:0) (Figure 2).

**Figure 3:**
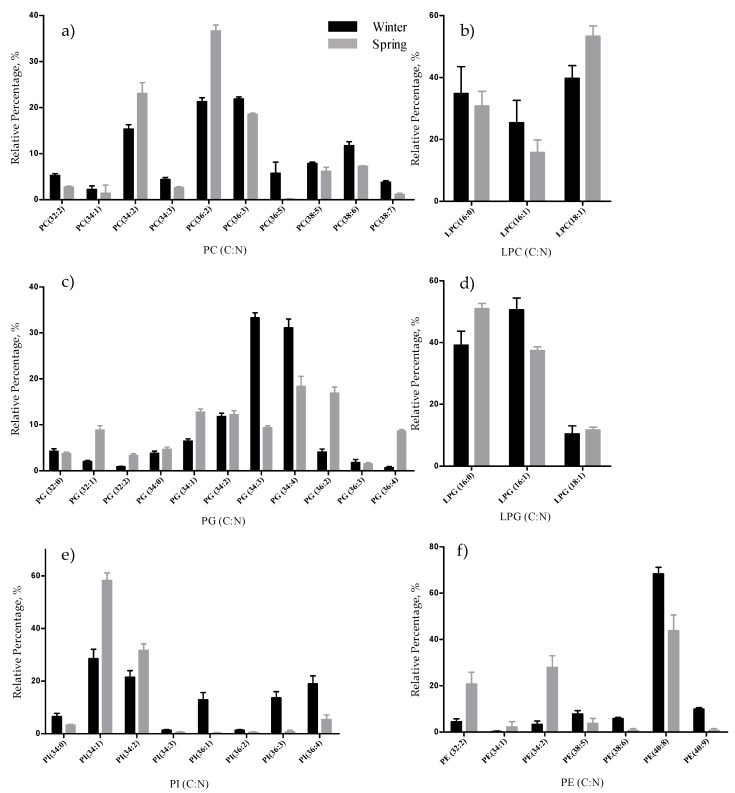
Relative abundance (RA) of molecular species of phospholipids identified in *F. vesiculosus* collected in winter (February) and spring (May), determined by HILIC LC-MS: (**a**) PC; (**b**) LPC; (**c**) PG; (**d**) LPG; (**e**) PI; and (**f**) PE molecular species. Numbers in parentheses (C:N) indicates the number of carbon atoms (C) and double bonds (N) in the fatty acid side chains. The species with higher relative abundance in each class were: PC (36:2) and PC (36:3), LPC (18:1), PG (34:4), PG (34:3), PG (36:2), LPG (16:1), PI (34:1), PI (34:2), and PE (40:8).

Regarding the phospholipids profile in *F. vesiculosus*, we identified 53 molecular species, distributed by the following classes: phosphatidylcholine (PC, 11 species), lyso-PC (LPC, 3 species), phosphatidylglycerol (PG, 16 species), lyso-PG (LPG, 3 species), phosphatidylinositol (PI, 9 species), lyso-PI (LPI, 1 species), phosphatidylethanolamine (PE, 8 species) and lyso-PE (LPE, 2 species) (Table 2, Supplementary information Figures S8 and S9). The PC and LPC species were identified in the mass spectra as [M + H]^+^ and [M + CH_3_COO]^−^ molecular ions and PE and LPE were identified as [M + H]^+^ and [M − H]^–^ ions. The remaining classes were assigned as [M − H]^–^ ions. The most abundant species in each class were: PC (36:2) and PC (36:3), LPC (18:1), PG (34:4), PG (34:3), PG (36:2), LPG (16:1), PI (34:1), PI (34:2), and PE (40:8) (Figure 3).

**Figure 4:**
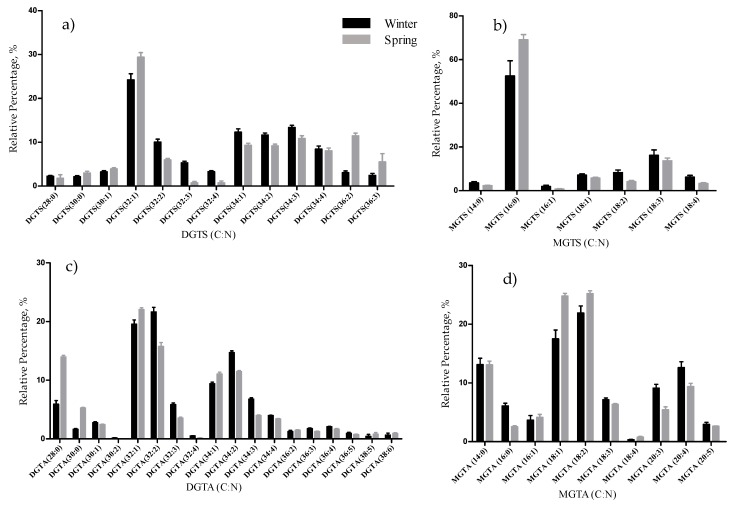
Relative abundance (RA) of molecular species of betaine lipids identified in *F. vesiculosus* collected in winter (February) and spring (May), determined by HILIC LC–MS: (**a**) DGTS; (**b**) MGTS; (**c**) DGTA; (**d**) MGTA molecular species. Numbers in parentheses (C:N) indicate the number of carbon atoms (C) and double bonds (N) in the fatty acid side chains. The species with higher relative abundance were DGTS (32:1), MGTS (16:0), DGTA (32:1), DGTA (32:2), MGTA(18:1), and MGTA (18:2).

**Figure 5:**
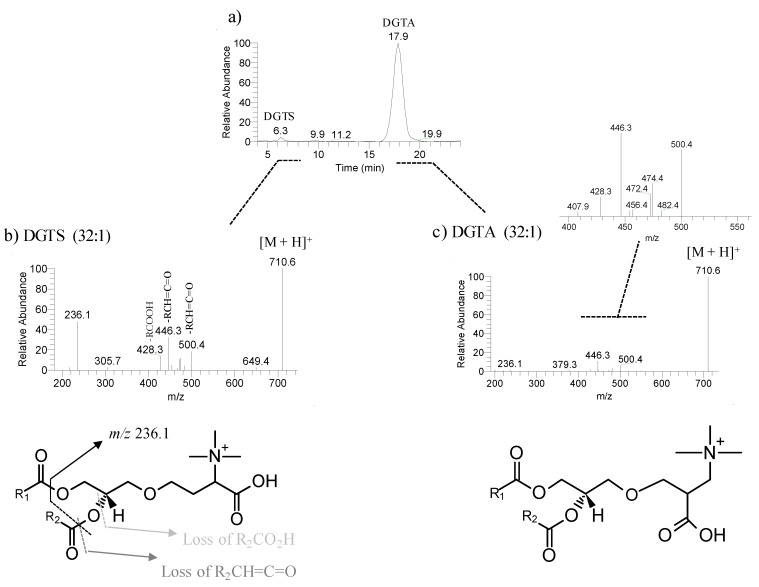
Reconstructed ion chromatogram (RIC) of the ions at m/z 710.6 identified as [M + H]^+^ ions DGTS (32:1) at a retention time (RT) of 6.3 min and DGTA (32:1) at RT of 17.9 min of a HILIC-LC-MS run of the total lipid extract of *F. vesiculosus* (**a**), and their respective MS/MS spectra (**b** and **c**). MS/MS spectra shows the product ions resultant from neutral loss of the fatty acyl chains as free fatty acid RCOOH and as ketene RCH=C=O and the typical product ion of betaine lipids at *m/z* 236.1 [37,38,39,40,41]. Inset in figure c) illustrates the magnification of the MS/MS spectrum of DGTA (32:1) and shows the product ions resultant from neutral loss of RCOOH and RCH=C=O, which are indicative of the fatty acyl composition.

We have also identified 53 molecular species of betaine lipids, distributed by diacylglyceryl hydroxymethyltrimethyl-β-alanine (DGTA, 21 species), and monoacylglyceryl hydroxymethyltrimethyl-β-alanine (MGTA, 10 species), and very low abundant diacylglyceryl-*N*,*N*,*N*-trimethyl-homoserine (DGTS, 15 species), and monoacylglyceryl-*N*,*N*,*N*-trimethyl-homoserine (MGTS, 7 species), (Table 3, Supplementary information Figure S6). These betaine lipids included molecular species with FA chain lengths ranging from C14 to C20 and the degree of unsaturations from 0 to 5. The most abundant molecular species were DGTA (32:1) and DGTA (32:2), MGTA (18:1); MGTA (18:2), DGTS (32:1) and MGTS (16:0) (Figure 4, Table 3). DGTA are well-known betaine lipids in *Fucus* sp., while DGTS are usually identified in the green and red macroalgae, as reported for example for *Codium tomentosum* [38], *Chondrus crispus* [37], *Gracilaria* sp. [39] and *Porphyra dioica* [40]. The classes DGTA and MGTA are structural isomers of DGTS and MGTS and were identified based on the LC retention time and in the manual interpretation of the MS/MS data (Supplementary information Figures S10–S12). The retention times were as follows: DGTA at RT = 16 min (Figure 5a), DGTS at RT = 6 min (Figure 5a), MGTS at RT = 9 min and MGTA at RT = 27 min (Supplementary information Figure S10).

### 2.3. Seasonal Polar Lipidome Profiles

We studied natural variation in the lipidome of *F. vesiculosus* in different abiotic conditions, from pooled samples collected in winter (February) and spring (May). Since there were significant differences in the abundances among different lipids and lipid classes, data sets were glog transformed and autoscaled to obtain the same weight of change for both low and high abundant lipids (Material and Methods, Section 4.6) and for comparability between different lipids for their biological relevance and used them in subsequent analyses. 

**Figure 6:**
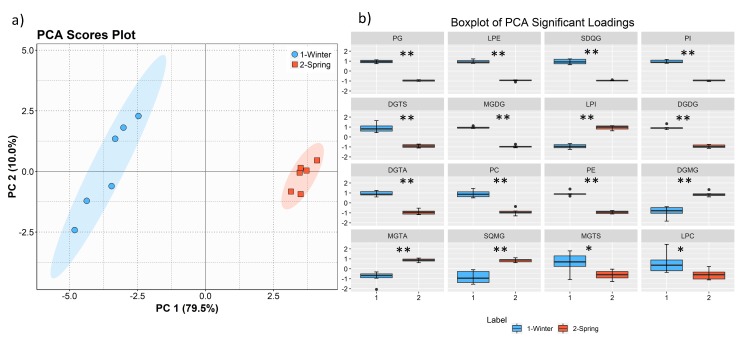
Principal component analysis (PCA) scores plot (PC2 vs. PC1) of the lipid data set acquired by LC-MS of the two groups: winter (February) and spring (May) (**a**) and boxplot of the lipid classes sorted (left to right, top to bottom) using the contributions in accounting for the variability in the first principal component of PCA (**b**). Mann–Whitney U test results displayed in box plots showing significant differences between seasons, * q < 0.05, ** q < 0.01.

A data matrix was constructed using the lipid classes, calculated from the sum of all molecular species within each class. The principal component analysis (PCA), showed clear discrimination of the two groups from the different seasons, with the eigenvalue of the first principal component representing 79.5% of the total variance (Figure 6a). We sorted lipid classes using the contributions of the significantly different variables in the first principal component, being the most important the PG, LPE, SDQG, and PI classes (Figure 6b).

**Figure 7:**
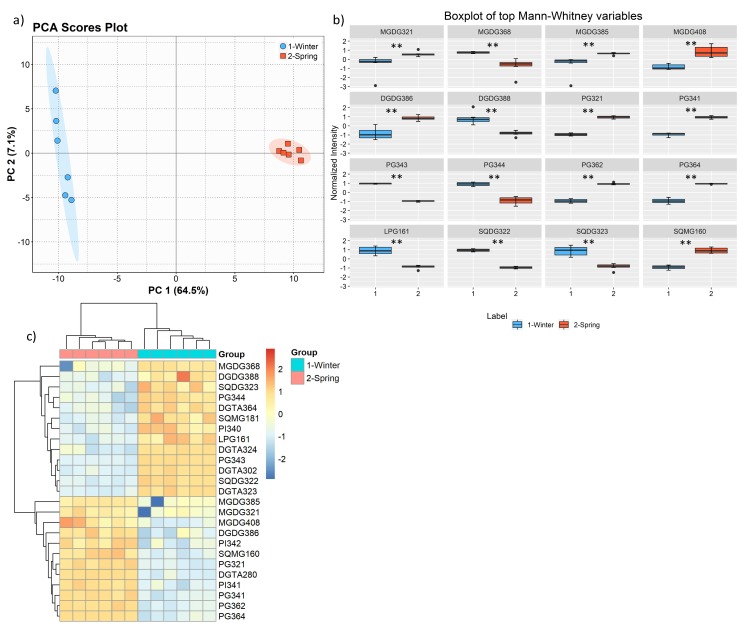
PCA score plot of two first PCs of lipid data set acquired by LC–MS of the two groups: winter (February) and spring (May) (**a**) and boxplot of the lipid species, sorted (left to right, top to bottom) using the q value. Mann–Whitney U test results displayed in box plots showing significant differences between seasons, ** q < 0.01. (**b**). Two-dimensional hierarchical clustering heat map of the polar lipid data (**c**). Levels of relative abundance are shown on the color scale, with numbers indicating the fold difference from the mean. The clustering of the sample groups is represented by the dendrogram in the top, showing two main clusters: February and May. The clustering of individual lipid species is represented by the dendrogram to the left, showing 2 main clusters. Labels of the species are according to the notation AAAAxxi (AAAA = lipid class; xx = total of carbon atoms in fatty acid; i = number of unsaturations).

The PCA of the lipid species dataset also showed clear discrimination of the two groups from different seasons (Figure 7a). The eigenvalues of the two first principal components represented 71.6% of the total variance (PC1 64.5% and PC2 7.1%) of the observations. Also, we sorted the molecular species using the q values from the Mann–Whitney U test. Results showed 107 molecular species with significant differences between seasons with q < 0.01 and 12 species with q < 0.05 (Supplementary Table S2). The top 16 molecular species with lower q values included 6 PG, 4 MGDG, 2 DGDG, 2 SQDG, 1 LPG, and 1 SQMG (Figure 7b). Of these, the species that were more abundant in May (spring) than in February were galactolipids MGDG (32:1), MGDG (38:5), MGDG (40:8), and DGDG (38:6), phospholipids PG (32:1), PG (34:1), PG 36:2, and PG 36:4, and sulfolipid SQMG (16:0). The species that were more abundant in February (winter) than in May (spring) were MGDG (36:8) and DGDG (38:6), phospholipids PG (34:3), PG (34:4), LPG 16:1 and sulfolipids SQDG (32:2), and SQDG (32:3).

We also used the information from the univariate analysis to create a dendrogram with a two-dimensional hierarchical clustering, using the top 25 p-values lipid species (q < 0.01) (Figure 7c). The primary split in the upper hierarchical dendrogram shows that the samples clustered independently in two groups, e.g., spring and winter. These two groups can be projected on the plane made by the first two dimentions of the PCA. The clustering of individual phospholipid species concerning their similarity in changes of lipid expression shows that they clustered in two main groups, one comprising 13 species that are more abundant in February, including 3 DGTA, 2PG, 2 DGDG, 2 SQDG, 1 MGDG, 1 SQMG, 1 LPG, 1 PI. The second group comprised 12 lipids that are more abundant in May and included 4 PG, 3 MGDG, 1 DGDG, 1 SQMG, 1 PI, and 1 DGTA. Remarkably, in the cold season, the species that showed higher abundance have higher unsaturation level and included PUFA (e.g., 18:3, 18:4, 20:5) while in the spring species that showed higher abundance included saturated, monounsaturated and PUFA (e.g., 18:2, 20:4) (Figure 7c).

#### Seasonal Total Fatty Acids Profiles

**Figure 8:**
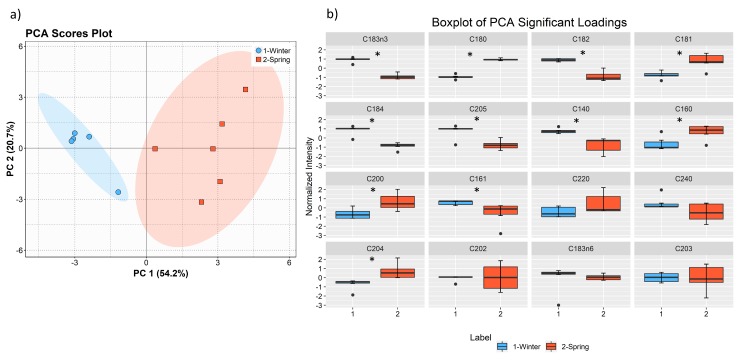
Principal component analysis (PCA) scores scatter plot (PC2 vs. PC1) of lipid (FA) data set acquired by GC-MS of the two groups: winter (February) and spring (May) (**a**) and boxplot of the FA, sorted (left to right, top to bottom) by contributions in accounting for the variability in the first principal component of PCA (**b**). Mann–Whitney U test results displayed in box plots showing significant differences between seasons, * q < 0.05.

The fatty acid composition in total lipids extracts from February and May batches of *F. vesiculosus* included sixteen fatty acids (Table 4), as previously reported [42,43]. *F. vesiculosus* from winter (February) and spring (May) contained the same total FA species, but with different relative amounts: PUFA 20:4(*n*-6) (10.7 ± 0.96% and 13.5 ± 1.81%, respectively), 18:2(*n*-6) (8.78 ± 0.22% and 6.62 ± 0.49%, respectively), 18:3(*n*-3) (7.13 ± 0.58% and 3.92 ± 0.34%, respectively) and 20:5(*n*-3) (6.69 ± 0.94% and 4.75 ± 0.47%, respectively). As described for the LC-MS analysis, in winter, *F. vesiculosus* contained higher amounts of PUFAs (40.0%), followed by SFA (31.7%) and MUFA (26.9%), and spring extracts contained SFA (35.6%), PUFAs (33.6%) and MUFAs (29.5%). Principal component analysis (PCA) of the FA data set also showed discrimination between the two groups (Figure 8a). The eigenvalues of the two first principal components represented 74.9% of the total variance (PC1 54.2% and PC2 20.7%) of the observations. We sorted FA using the contributions of the significantly different variables in the first principal component, being the most important the C18:3*n*-3, C18:0, C18:2, and C18:1 FA (Figure 8b). Of the 16 FA that were quantified, 11 were significantly different (q<0.05): FA 18:3(*n*-3), 18:2(*n*-6), 18:4(*n*-3), 20:5(*n*-3) and the saturated FA 14:0 were more abundant in winter, while FA 16:0, 18:0, 18:1 and 20:4(*n*-6) were more abundant in the FA spring.

### 2.4. Degree of Unsaturation of Membrane Lipids

The double bond index (DBI) was calculated for the FA and the polar lipid profiles (Table 5). This index measures the average number of double bonds in the fatty acids esterified to polar lipid molecular species, indicating the level of unsaturation. Considering the FA profile from *F. vesiculosus*, the DBI in February was higher, indicating a higher average number of double bonds in the esterified fatty acids. Generally, the DBI of the polar lipid classes also shows a higher DBI in February samples, although differences are observed between classes. Higher variations in DBI (expressed in RC%) between seasons were observed in the extra-plastidial classes lipids, PE, PI, and PC, in the glycolipids SQDG and SQMG and in DGTA betaine lipids.

## 3. Discussion

In this work, the profile in the polar lipid of *F. vesiculosus* was identified for the first time using a modern high-resolution LC–MS platform. We have identified in the lipidome of *F. vesiculosus* eighteen different classes of polar lipids such as the glycolipids MGDG, MGMG, DGDG, DGMG, SQDG, and SQMG, the phospholipids PC, LPC, PE, LPE, PG, LPG, PI, and LPI and the betaine lipids DGTA, MGTA, DGTS; and MGTS, in a total of 187 molecular species. It is well known that the lipid composition and dynamics of the algae lipidome is modulated by different environmental conditions, such as temperature and light, as usually experienced in different seasons or geographical origins [33,44]. Herein, we evaluated the plasticity of the lipidome of *Fucus* in two seasons, February (winter) and May (spring). In that year, winter´s batch temperature and light expositions were 13.2 ± 1.17 °C and 177 µmol photons m^2^ s^−1^ (average irradiance), and in the spring were 17.4 ± 1.36 °C and 424 µmol photons m^2^ s^−1^, respectively.

The total lipid content was significantly higher in the winter than in the spring (1.74% versus 1.47% DW), although we have identified the same polar lipids species in samples from both seasons. We observed significant differences in the relative abundances of several polar lipids classes and molecular species (Figure 1, Figure 2, Figure 3, Figure 4, Figure 5, Figure 6 and Figure 7, Table 5), but also in several fatty acids from the total lipid extracts (Figure 8). There was an increase of the relative abundance (RA) of lyso-glycolipids and lyso-betaine lipids in May, while diacyl polar lipids had higher RA in the winter samples. Also, samples from the winter season were more abundant in glycolipids and phospholipids containing highly unsaturated FA and in betaine lipids combining short chain acid saturated FA and PUFA. Similarly, the glycolipids of samples from winter had higher RA of species containing long-chain FA with degrees of unsaturation from 3 to 5 double bonds, when compared with samples from spring. This tendency was previously reported for *F. serratus* [33], that in the winter had increased total percentage of PUFA and polar lipids rich in PUFA (MGDG, PG, and DGDG). Such seasonal changes in the polar lipid composition were associated by the authors with temporal changes in winter (low light intensity, low irradiance, and shorter day length). Under low light intensity, the RA of the glycolipids from chloroplast membranes (MGDG, DGDG, SQDG) increased most probably to adaptation of the thylakoids membranes and the maintenance of photosynthetic processes [45,46,47,48]. The ability of algae to adjust the ratio MGDG/DGDG composition has been suggested to be a strategy in coping with the low temperature [33,49], and the combined effect of the increased level of unsaturations of glycolipids is considered crucial for the stabilization of the photosynthetic apparatus and of proteins in the membrane. Also, the increase of the RA of the SQDG class could be related to its protective effect on the photosynthetic protein complexes [33]. The plasticity of phospholipids to weather seasons also followed this trend in the acclimatization to the winter season, characterized by higher RA of unsaturated FA, mainly due to the contribution of PG. PG has an important role associated with the photosynthetic membrane preservation, which can be comparable to SQDG [33].

In this study, DGTA were the most abundant betaine lipids, as in previous reports that considered this betaine class a taxonomic marker of brown macroalgae [50,51]. DGTA are found exclusively in the brown macroalgae while DGTS are typically present in the green and red macroalgae. The plasticity and adaptation to environmental change of betaine lipids were evidenced by the increase of molecular species with a longer chain length and unsaturated FA in the winter samples. The role of betaine lipids in response to seasonal changes has not been reported yet. Nevertheless, in the summer, DGTA was found to be overexpressed in *F. vesiculosus*, with higher RA of saturated molecular species such as DGTA 28:0 and 30:0 [26]. However, the authors did not find a relation between unsaturation and the seasonality pattern, as we describe it in this study.

The trend we have observed for the increase of FA unsaturation in the winter season was corroborated either by the polar lipidomics data and the total FA data and further evidenced by using the Double bond index (DBI) analysis. The adaptation of the lipid metabolism in brown macroalgae to lower temperatures during winter, by modulating the biosynthesis of PUFA and increasing lipid desaturation, was already reported in studies that evaluated the fatty acid profile of *F. vesiculosus* [33,52,53]. A similar effect was observed in the brown macroalgae *Undaria pinnafida* [29], *Sargassum pallidium* [28], *Egregia menziesii* [32], and *Costaria costata* [54] that showed higher content of PUFA and higher *n*-3/*n*-6 ratio in response to cold temperatures. The proportion of saturated and unsaturated acyl chains in membrane lipids is a critical factor that affects lipid packing, membrane viscosity, and water permeability. In Poikilothermic organisms, such as algae, that do not control their body temperature, the increase the proportion of unsaturated acyl chains in membrane lipids is essential to maintain fluidity at lower temperatures and to hold photosynthesis [49].

Herein, we have also profiled the total fatty acids, including those esterified to polar lipid in *F. vesiculosus,* and find that included saturated, monounsaturated and polyunsaturated FA, with a predominance of omega 3 PUFA 18:3, 18:4 and 20:5. As observed in the polar lipid composition determined by LC–MS/MS, omega 6 PUFA, namely 18:2 and 20:4, were also abundant. The calculated ratio of *n*-6/*n*-3 FA was 1.20 in winter and 1.95 in spring, with an average of 1.57, which is a lower ratio than the maximum recommended by the World Health Organization (lower than 10) [55,56]. As such, *F. vesiculosus* can be considered a sustainable source of PUFA [28,29,51,57], either available by the consumption as edible seaweed, or used as a raw material in food industries and biotechnological applications [4]. Due to its content in *n*-6 and *n*-3 PUFA, the consumption of this seaweed could have benefits for preventing cardiovascular disorders, depression and other mental disorders [56]. Despite the observed variations in the RA of polar lipids and total FA, the molecular species bearing PUFA were still the most abundant ones in both seasons, and thus both batches have a remarkable composition to be used for food or feed diet as a source of lipids with nutritional value.

Phospholipids can also be explored as add value ingredients. They are considered appealing food ingredients, for fortification of the content of foods in *n*-3 FAs such as 20:5 (*n*-3) and to enhance its nutritional values [58]. There are also recognized health benefits in the consumption of PL bearing to *n*-6 and *n*-3 PUFA, contributing to maintain normal physiological functions and to prevent inflammatory diseases, among others. This foster the growing interests in the use of PLs from macroalgae in the nutraceutical industries [58,59,60]. Polar lipids are also claimed for cosmetics and pharmaceutical applications, namely phospholipids used as carriers liposomes, lipid emulsions, micelles, drug-phospholipids complexes and cochleates [61,62].

The polar lipidome of *F. vesiculosus* also included some species that were reported as bioactive phytochemicals. The glycolipids MGDG (20:5/18:3) and MGDG (20:5/18:4), that in this study were abundant species in both seasons, were previously isolated from *Fucus spiralis* [24]. These species showed anti-inflammatory activity, as they reduced the NO release by activated macrophages in a dose-dependent manner [24]. Furthermore, MGDG (18:4/16:0), DGDG(18:4/16:0), DGDG (20:4/16:0), DGDG (20:5/16:0) and SQDG (20:5/14:0), also identified in this study, were associated with anti-inflammatory activity through down-regulation of iNOS [63]. Also, the MGDG (20:5/18:4) was found to inhibit the growth of human melanoma cells [25]. Antimicrobial activity was associated with MGDG (20:5/18:4) and MGDG (18:3/18:4) molecular species [64] and also SQDG molecular species [22]. Sulfolipids, such as SQDG or SQMG, which in this study increased in the spring samples, were associated with anti-HSV1 and anti-HSV2 activities [25] while SQMG isolated from the extract of the brown macroalga was active against *Xanthomonas oryzae* pv. Oryzae and associated with antibacterial activity [65]. Glycolipids can also be ingredients in cosmetic formulations and as biosurfactants, highly considered to replace synthetic surfactants [66].

The composition in lipid herein assigned to *F. vesiculosus* supports its use as a functional food or as raw biomass, source of polar lipids, to be used as food ingredients, or even as a source of bioactive lipids. Our results on the lipidomics profile of *F. vesiculosus* in two different seasons contribute to a better understanding of the lipidome plasticity and adaptation to environmental changes and reinforces the importance on the knowledge of the lipidome at a molecular level for bioprospection of the potential of brown macroalgae as a source of natural products for different biotechnological applications.

## 4. Materials and Methods

### 4.1. Reagents

HPLC grade chloroform, methanol, and acetonitrile were purchased from Fisher Scientific Ltd. (Loughborough, UK). All other reagents were purchased from major commercial sources. Milli-Q water (Synergy, Millipore Corporation, Billerica, MA, USA) was used.

### 4.2. Biomass

Macroalgae were provided by ALGAplus Ltd. (production site in Ria de Aveiro, mainland Portugal, 40°36′43″N, 8°40′43″W). *F. vesiculosus* was harvested within the margins of the fish ponds and the exit channels of the aquaculture site. Before processing for food, the seaweed was maintained in tanks with controlled conditions for two weeks. *F. vesiculosus* samples were collected on February 2016 representing the winter—F1.0716M, and on May 2016, spring season—F1.2116D. February batch was maintained at 13.2 ± 1.17 °C, average salinity 27.0 PSU, and an average irradiance of 177 µmol photons m^2^ s^−1^; May batch was cultivated at 17.4 ± 1.36 °C, average salinity of 27.5, and average irradiance of 424 µmol photons m^2^ s^−1^. Samples/batches were obtained from bulk production. The biomass was cleaned to remove epiphytes and oven-dried at 25 °C (up to 12% moisture content). Aliquots of biomass of 250 mg were obtained from bulk production and were used to extract total lipids. Extraction procedure was done at least in triplicate.

### 4.3. Lipid Extraction

Total lipid extraction was performed by adding methanol/chloroform (2:1, per volume) to three replicate samples of 250 mg of seaweed per each season, as already described in previous studies performed by the research team [37,39,41]. The mixture was transferred to a glass tube with a Teflon-lined screw cap and, after addition of 3.75 mL of a mixture methanol/chloroform (2:1, per volume), was homogenized and incubated on ice on a rocking platform shaker (Stuart Scientific STR6, Bibby, UK) for 2 h and 30 min. The mixture was centrifuged at 2000 rpm for 10 min, and the organic phase was collected. The biomass residue was re-extracted three times with 1 mL of MeOH and 0.5 mL of CHCl_3_. Water was added to the total collected organic phase, followed by centrifugation for 10 min at 2000 rpm, and the organic (lower) phase was recovered. Solvents were dried under a stream of nitrogen gas. The total lipid extract content was estimated by gravimetry. Lipid extracts were stored at –20 °C before analysis by LC-MS and GC-MS.

### 4.4. Polar Lipid Analysis by HILIC−LC−Q−Exactive-MS

High-performance LC (HPLC) system (Thermo scientific AccelaTM, Waltham, MA, USA) with an autosampler coupled online to the Q-Exactive® mass spectrometer with Orbitrap® technology was used. The solvent system consisted of two mobile phases as follows: mobile phase A (acetonitrile:methanol:water 50:25:25 (per volume) with 1 mM ammonium acetate) and mobile phase B (acetonitrile:methanol 60:40 (per volume) with 1 mM ammonium acetate). Initially, 0% of mobile phase A was held isocratically for 8 min, followed by a linear increase to 60% of A within 7 min and a maintenance period of 15 min, returning to the initial conditions in 10 min. A volume of 5 µL of each sample containing 5 µg of lipid extract, a volume of 4 μL of phospholipid standards mix (dMPC - 0.02 μg, dMPE - 0.02 μg, NPSM - 0.02 μg, LPC - 0.02 μg, dPPI - 0.08 μg, dMPG - 0.012 μg, dMPS - 0.04 μg) and 91 µL of eluent B was introduced into the Ascentis® Si column (15 cm × 1 mm, 3 µm, Sigma-Aldrich) with a flow rate of 40 µL min^–1^ and at 30 °C.

The mass spectrometer with Orbitrap® technology was operated simultaneously in positive (electrospray voltage 3.0 kV) and negative (electrospray voltage –2.7 kV) modes with a resolution of 70,000 and automatic gain control (AGC) target of 1 × 10^6^, the capillary temperature was 250 °C and the sheath gas flow was 15 U. In MS/MS experiments, a resolution of 17,500 and AGC target of 1 × 10^5^ were used. Cycles consisted of one full scan mass spectrum and ten data-dependent MS/MS scans were repeated continuously throughout the experiments with the dynamic exclusion of 60 s and intensity threshold of 1 × 10^4^. Normalized collision energy™ (CE) ranged between 25, 30 and 35 eV. Data acquisition was carried out using the Xcalibur data system (V3.3, Thermo Fisher Scientific, Waltham, MA, USA). Six replicates were performed, corresponding to two analytical replicates of three lipid extracts (total of six replicates, N = 2 × 3). The identification of molecular species of polar lipids was based on the LC retention time (Supplementary information Figures S1, S2, S6, S7, S10), mass accuracy (Supplementary information Table S1) and detailed structural information inferred by MS/MS data (Supplementary information Figures S3–S5, S8–S9, S11, S12). Accurate mass measurements (≤5 ppm) were used to confirm the elemental composition calculation of empirical formula. Structural characterization of molecular species was based on tandem mass spectra interpretation to confirm polar head group and fatty acyl chains [37,38,39,40,41,67].

### 4.5. Fatty Acid Analysis by GC−MS

Fatty acid methyl esters (FAMEs) were prepared from the total lipid extracts using a methanolic solution of potassium hydroxide (2.0 M) according to the methodology used in our laboratory [37]. Sample volumes of 2.0 μL of the hexane solution containing FAMEs were analyzed by gas chromatography-mass spectrometry (GC–MS) on an Agilent Technologies 6890 N Network (Santa Clara, CA, USA) equipped with a DB–FFAP column with the following specifications: 30 m of length, 0.32 mm internal diameter, and 0.25 μm film thickness (123-3232, J&W Scientific, Folsom, CA, USA). The GC equipment was connected to an Agilent 5973 Network Mass Selective Detector operating with an electron impact mode at 70 eV and scanning the range *m/z* 50–550 in a 1 s cycle in a full scan mode acquisition. The oven temperature was programmed from an initial temperature of 80 °C for 3 min; a linear increase to 160 °C at 25 °C min^−1^; a linear increase at 2 °C min^−1^ to 210 °C; and a linear increase at 30 °C min^−1^ to 250 °C followed by 10 min at this temperature. Helium was used as carrier gas at a flow rate of 1.4 mL min^−1^. Two analytical replicates of each of the three lipid extracts (total of six replicates, N = 2 × 3) were performed. The identification of each FA was performed considering the retention times and comparison with MS spectra of FA standards (Supelco 37 Component Fame Mix, Sigma-Aldrich, St. Louis, MO, USA) and those in the Wiley 275 library and AOCS Lipid Library. The relative amounts of FAs were calculated by the percent relative area method with proper normalization using internal standard, methyl nonadecanoate (C19:0, Sigma-Aldrich, St. Louis, MO, USA), and considering the sum of all relative areas of the identified FAs. Results were expressed as means ± SD.

### 4.6. Data Processing and Statistical Analysis

The MS raw data were pre-processed by using the software package MZmine 2 with a mass tolerance of 5 ppm. Data integration was expressed by the changes of the relative abundance of molecular species of all classes, previously normalized against selected internal standards. The results were expressed as a percentage, obtained by dividing the normalized peak areas of each molecular species by the sum of total peak areas. The double bond index was calculated as DBI = Σ (weight % of the fatty acids × N)/100 [68], and for the lipid class it was calculated as DBI = Σ (relative percentage of lipids × N)/100, N corresponded to the number of double bonds of each unsaturated fatty acid [49].

Multivariate and univariate analyses were performed using R version 3.5.1 [69] in Rstudio version 1.1.4 [70]. GC data were glog transformed, and HPLC/MS data were glog transformed and autoscaled using the R package Metaboanalyst [71]. Principal Component Analysis (PCA) was conducted for exploratory data analysis, with the R built-in function and ellipses were drawn using the R package ellipse [72], assuming a multivariate normal distribution and a level of 0.95. Mann-Whitney U test comparisons with Benjamini-Hochberg correction for multiple hypothesis testing with a cutoff false discovery rate (FDR) value of 5% were performed with the R built-in function. Statistical differences were calculated and represented with the following symbols of significance level **, significantly different q < 0.01, * significantly different q < 0.05. Heatmaps were created using the R package pheatmap [73] using "Euclidean" as clustering distance, and "ward.D" as the clustering method. All graphics and boxplots were created using the R package ggplot2 [74]. Other R packages used for data management and graphics included plyr [75], dplyr [76], and tidyr [77].

## 5. Conclusions

This work showed new insights into the identification of the lipidome of *F. vesiculosus* at a molecular level. A total of 187 molecular species distributed by glycolipids, phospholipids, and betaine lipids were identified. The lyso-betaine MGTA is reported for the first time in macroalgae. The lipidome plasticity and adaptation to environmental changes with the season (winter and spring) of *F. vesiculosus* was evidenced by the variation in the content of lipids bearing MUFA and PUFA. Multivariate statistical analysis, PCA and cluster analysis, showed a clear clustering according to lipid diversity and seasonal groups. In winter (February), the effects of lower temperature and lower light exposition period lead to the increase of polyunsaturated esterified FA majorly of glycolipids, some phospholipid and DGTA betaine lipid classes.

*F. vesiculosus* lipidome contained eighteen classes of polar lipids that were the main source of the 18:3(*n*-3), 18:4(*n*-3), 20:4(*n*-6), and 20:5(*n*-3) PUFA, with high nutritional value. The low *n*-6/*n*-3 ratio renders *F. vesiculosus* suitable for human consumption and feed, independent of the seasonal variation (February and May collections). Also, PUFA bearing polar lipid species that were reported with important bioactive properties were identified in the lipdome of *Fucus*, with relevance for the anti-inflammatory, antitumoral and antimicrobial properties of some lipid species, fostering the use of *F. vesiculosus* as a suitable raw material of active ingredients. Noteworthy, the glycolipids species more abundant, mainly in the winter lipidome, were reported to have anti-inflammatory and antitumoral properties. Whereas, sulfolipids SQDG and SQMG species, more abundant in the spring, were associated with antimicrobial activity.

## Figures and Tables

**Table 1:** Glycolipids identified by HILIC LC–MS and MS/MS in *F. vesiculosus* (mass error < 5 ppm). MGDG and DGDG molecular species were identified as [M + NH_4_]^+^ ions while SQDG and SQMG were identified as [M – H]^−^ ions. Numbers in parentheses (C:N) indicate the number of carbon atoms (C) and double bonds (N) in the fatty acid side chains.

**Galactolipids [M + NH_4_]^+^**		
**Observed *m/z***	**Lipid Species (C:N)**	**Fatty Acyl Chain**
Monogalactosyl diacylglycerol - MGDG		
746.5765	MGDG (32:1)	18:1/14:0, 16:1/16:0
768.5621	MGDG (34:4)	18:4/16:0, 18:3/16:1
770.5764	MGDG (34:3)	18:3/16:0
772.5935	MGDG (34:2)	18:2/16:0, 18:1/16:1
774.6074	MGDG (34:1)	18:1/16:0
788.5307	MGDG (36:8)	18:4/18:4
790.5459	MGDG (36:7)	18:3/18:4
792.5614	MGDG (36:6)	18:3/18:3
794.5772	MGDG (36:5)	18:2/18:3, 20:5/16:0
796.5929	MGDG (36:4)	20:4/16:0, 18:2/18:2
814.5449	MGDG (38:9)	20:5/18:4
816.5608	MGDG (38:8)	20:5/18:3, 20:4/18:4
818.5766	MGDG (38:7)	20:5/18:2, 20:4/18:3
820.5926	MGDG (38:6)	20:5/18:1, 20:4/18:2
822.6073	MGDG (38:5)	20:4/18:1
824.6235	MGDG (38:4)	20:4/18:0
842.5751	MGDG (40:9)	20:5/20:4
844.594	MGDG (40:8)	20:4/20:4
Monogalactosyl monoacylglycerol - MGMG		
536.3795	MGMG (18:1)	
530.3329	MGMG (18:4)	
532.3488	MGMG (18:3)	
534.3642	MGMG (18:2)	
Digalactosyl diacylglycerol - DGDG		
906.6148	DGDG (32:2)	14:0/18:2
908.6305	DGDG (32:1)	14:0/18:1, 16:0/16:1
930.6135	DGDG (34:4)	*
932.6305	DGDG (34:3)	18:3/16:0
934.6461	DGDG (34:2)	18:2/16:0, 18:1/16:1
936.6618	DGDG (34:1)	18:1/16:0
938.678	DGDG (34:0)	18:0/16:0
952.5992	DGDG (36:7)	18:3/18:4
954.6148	DGDG (36:6)	18:3/18:3
956.6305	DGDG (36:5)	20:5/16:0, 18:2/18:3
958.6461	DGDG (36:4)	20:4/16:0, 18:2/18:2
960.6623	DGDG (36:3)	18:1/18:2
962.678	DGDG (36:2)	18:1/18:1
964.6936	DGDG (36:1)	18:1/18:0
976. 5997	DGDG (38:9)	20:5/18:4
978.6144	DGDG (38:8)	20:5/18:3, 20:4/18:4
980.6289	DGDG (38:7)	20:4/18:3, 20:5/18:2
982.6448	DGDG (38:6)	20:4/18:2, 20:5/18:1
984.6594	DGDG (38:5)	20:4/18:1
986.6767	DGDG (38:4)	20:4/18:0
Digalactosyl monoacylglycerol - DGMG		
672.417	DGMG (16:0)	
692.3857	DGMG (18:4)	
694.4014	DGMG (18:3)	
696.417	DGMG (18:2)	
698.4327	DGMG (18:1)	
**Sulfolipids [M – H]^–^**		
Sulfoquinovosyl monoacylglycerol - SQMG		
527.2515	SQMG (14:0)	
553.2664	SQMG (16:1)	
555.2829	SQMG (16:0)	
577.2673	SQMG (18:3)	
579.2832	SQMG (18:2)	
581.2984	SQMG (18:1)	
Sulfoquinovosyl diacylglycerol - SQDG		
737.4495	SQDG (28:0)	14:0/14:0
763.4648	SQDG (30:1)	16:0/14:1
765.4804	SQDG (30:0)	16:0/14:0
787.4646	SQDG (32:3)	18:3/14:0
789.4804	SQDG (32:2)	18:2/14:0
791.4958	SQDG (32:1)	18:1/14:0
793.5099	SQDG (32:0)	18:0/14:0
813.4803	SQDG (34:4)	18:3/16:1
815.4958	SQDG (34:3)	18:3/16:0
817.5107	SQDG (34:2)	18:2/16:0
819.5269	SQDG (34:1)	18:1/16:0
839.4977	SQDG (36:5)	20:5/16:0
841.5106	SQDG (36:4)	20:4/16:0

**Table 2:** Phospholipid identified by HILIC LC–MS and MS/MS in *F. vesiculosus* (mass error < 5 ppm). PC and LPC molecular species were identified as [M + H]^+^ ions while PG, LPG, PI, LPI, PE, and LPE were identified as [M – H]^–^ ions. Numbers in parentheses (C:N) indicates the number of carbon atoms (C) and double bonds (N) in the fatty acid side chains.

**Phospholipids [M + H]^+^**		
**Observed *m/z***	**Lipid species (C:N)**	**Fatty acyl chain**
Phosphatidylcholine - PC		
730.538	PC (32:2)	16:1/16:1
756.55	PC (34:3)	16:1/18:2
758.5689	PC (34:2)	16:0/18:2, 16:1/18:1
760.5835	PC (34:1)	
780.553	PC (36:5)	18:2/18:3
784.585	PC (36:3)	18:1/18:2
786.6005	PC (36:2)	18:1/18:1
804.5545	PC (38:7)	
806.5683	PC (38:6)	
808.5831	PC (38:5)	
Lyso-phosphatidylcholine - LPC		
494.3241	LPC (16:1)	
496.3392	LPC (16:0)	
522.3556	LPC (18:1)	
**Phospholipids [M − H]^−^**		
**Observed *m/z***	**Lipid species (C:N)**	**Fatty acyl chain**
Phosphatidylglycerol - PG		
717.471	PG (32:2)	16:0/16:2, 16:1/16:1
719.4868	PG (32:1)	16:1/16:0, 14:0/18:1
721.5033	PG (32:0)	16:0/16:0, 14:0/18:0
741.4713	PG (34:4)	16:1/18:3
743.4862	PG (34:3)	16:0/18:3, 16:1/18:2
745.5018	PG (34:2)	16:0/18:2
747.5183	PG (34:1)	16:1/18:0
749.5324	PG (34:0)	16:0/18:0
769.4987	PG (36:4)	16:0/20:4, 18:2/18:2
771.516	PG (36:3)	18:1/18:2
773.5321	PG (36:2)	18:1/18:1
Lyso-phosphatidylglycerol - LPG		
481.2569	LPG (16:1)	
483.2733	LPG (16:0)	
509.2887	LPG (18:1)	
Phosphatidylinositol - PI		
831.4984	PI (34:3)	16:0/18:3
833.5175	PI (34:2)	16:0/18:2
835.5339	PI (34:1)	16:0/18:1
837.5491	PI (34:0)	16:0/18:0
857.5196	PI (36:4)	16:0/20:4
859.5352	PI (36:3)	18:0/18:3
861.5505	PI (36:2)	18:0/18:2, 18:1/18:1
863.5674	PI (36:1)	18:0/18:1
Lyso-Phosphatidylinositol - LPI		
597.306	LPI (18:1)	
Phosphatidylethanolamine		
686.4758	PE (32:2)	14:0/18:2, 16:1/16:1
714.5067	PE (34:2)	16:1/18:1
716.5227	PE (34:1)	16:0/18:1
762.5073	PE (38:6)	18:2/20:4
764.5232	PE (38:5)	18:1/20:4
784.4918	PE (40:9)	20:4/20:5
786.5076	PE (40:8)	20:4/20:4
Lyso-Phosphatidylethanolamine - LPE		
498.2613	LPE (20:5)	
500.2779	LPE (20:4)	

**Table 3:** Betaine lipids identified by HILIC LC–MS and MS/MS in *F. vesiculosus* (mass error < 5 ppm). DGTS, MGTS, DGTA, and MGTA molecular species were identified as [M + H]^+^ ions. Numbers in parentheses (C:N) indicate the number of carbon atoms (C) and double bonds (N) in the fatty acid side chains, ** with the contribution of sodium adducts.

**Betaine lipids [M + H]^+^**		
**Observed *m/z***	**Lipid species (C:N)**	**Fatty acyl chain**
Diacylglyceryl trimethyl-homoserine - DGTS		
656.5468	DGTS (28:0)	14:0/14:0
682.5628	DGTS (30:1)	14:0/16:1
684.5787	DGTS (30:0)	14:0/16:0
704.5458	DGTS (32:4)	16:0/16:4
706.5617	DGTS (32:3)	14:0/18:3, 16:0/16:3
708.5778	DGTS (32:2)	16:0/16:2
710.5933	DGTS (32:1)	14:0/18:1, 16:0/16:1
732.5774	DGTS (34:4)	16:0/18:4
734.5932	DGTS (34:3)	16:0/18:3
736.6083	DGTS (34:2)	16:0/18:2
738.624	DGTS (34:1)	16:0/18:1
762.6248	DGTS (36:3)	18:0/18:3
764.6405	DGTS (36:2)	18:0/18:2
Monoacylglyceryl trimethyl-homoserine - MGTS		
446.347	MGTS (14:0)	
472.3632	MGTS (16:1)	
474.3792	MGTS (16:0)	
494.3477	MGTS (18:4)	
496.3634	MGTS (18:3)	
498.3796	MGTS (18:2)	
500.3943	MGTS (18:1)	
Diacylglyceryl hydroxymethyl-trimethyl-β-alanine - DGTA		
656.547	DGTA (28:0)	14:0/14:0
680.5433	DGTA (30:2)	
682.5623	DGTA (30:1)	14:0/16:1
684.5782	DGTA (30:0)	14:0/16:0
704.5464	DGTA (32:4)	14:0/18:4
706.5625	DGTA (32:3)	14:0/18:3
708.5778	DGTA (32:2)	14:0/18:2
710.5933	DGTA (32:1)	14:0/18:1, 16:0/16:1
732.5776	DGTA (34:4)	**14:0/20:4
734.5935	DGTA (34:3)	14:0/20:3, 16:0/18:3
736.6091	DGTA (34:2)	14:0/20:2, 16:0/18:2
738.6241	DGTA (34:1)	16:0/18:1
758.5925	DGTA (36:5)	16:0/20:5
760.6085	DGTA (36:4)	**16:0/20:4
762.6246	DGTA (36:3)	16:0/20:3
764.6406	DGTA (36:2)	18:1/18:1
784.6084	DGTA (38:6)	18:2/20:4
786.6235	DGTA (38:5)	18:1/20:4
Monoacylglyceryl hydroxymethyl-trimethyl-β-alanine - MGTA		
446.3464	MGTA (14:0)	
472.3618	MGTA (16:1)	
474.3777	MGTA (16:0)	
494.3463	MGTA (18:4)	
496.3623	MGTA (18:3)	
498.3778	MGTA (18:2)	
500.3941	MGTA (18:1)	
520.3619	MGTA (20:5)	
522.3779	MGTA (20:4)	
524.3958	MGTA (20:3)	

**Table 4:** Fatty acid profile of *F. vesiculosus* collected in winter and spring, determined by GC–MS analysis of fatty acid methyl esters (FAMEs). AA, Arachidonic acid; EPA, Eicosapentaenoic acid; Means (%) and standard deviations (S.D.) were obtained from six replicates. Uns., unsaturated; sat. saturated; Others, FAMEs with relative abundance < 0.5% (FA C15:0, C16:2, C16:3, C16:4, C17:0, C17:1, C20:1).

	Winter		Spring	
**Fatty acids**	**Mean (%)**	**±SD**	**Mean (%)**	**±SD**
14:0	10.9	1.08	6.58	1.48
16:0	17.2	1.98	20.8	2.12
16:1*n*-7	1.55	0.05	1.41	0.1
18:0	1.64	0.25	6.09	0.43
18:1	25.3	0.69	28.1	1.44
18:2*n*-6	8.78	0.22	6.62	0.49
18:3*n*-6	0.89	0.41	0.7	0.11
18:3*n*-3	7.13	0.58	3.92	0.34
18:4*n*-3	4.35	0.51	2.71	0.19
20:0	0.35	0.11	0.59	0.14
20:2*n*-6	0.71	0.04	0.7	0.15
20:3*n*-6	0.71	0.09	0.7	0.17
20:4*n*-6	10.7	0.96	13.5	1.81
20:5*n*-3	6.69	0.94	4.75	0.47
22:0	0.5	0.07	0.65	0.25
24:0	1.11	0.23	0.90	0.22
Others	1.49		1.30	
**Σ SFA**	**31.7**		**35.6**	
**Σ** **ΜUFA**	**26.9**		**29.5**	
**Σ PUFA**	**40.0**		**33.6**	
AA/EPA	1.60		2.84	
**Σ***n*−6/**Σ***n*−3	1.20		1.95	
**Ratio (Unsat/Sat)**	2.11		1.77	

**Table 5:** Double bond index (DBI) of membrane lipids in *F. vesiculosus* collected in February and in May. The relative change (RC) in DBI from winter (February) to spring (May) calculated as RC = ((DBI_February_ − DBI_May_)/DBI_February_) × 100. The FA double bond index was calculated as DBI = Σ (weight % of the fatty acids × N)/100 and for the lipid class was calculated as DBI = Σ (relative percentage of lipids × N)/100, being N = number of double bonds of each FA. The relative percentage of means were calculated considering classes with more than three species.

Lipid Class	DBI		
	Winter	Spring	RC (%)
MGDG	6.90	5.89	15%
MGMG	2.75	2.75	0.0%
DGDG	5.70	5.36	6.3%
DGMG	2.57	2.12	18%
SQDG	1.33	1.00	25%
SQMG	1.35	1.10	19%
PI	2.07	1.47	29%
PC	3.32	2.70	19%
PG	2.74	2.27	17%
PE	7.20	4.80	33%
DGTS	1.94	1.77	9.0%
MGTS	1.67	1.49	11%
DGTA	1.89	1.54	19%
MGTA	2.05	1.79	13%
Fatty acids	1.66	1.53	7.8%

## Supplementary Materials

The following are available online at https://www.mdpi.com/1660-3397/17/6/335/s1.

## Abbreviations

DBI	Double bond Index
DGDG	Digalactosyl diacylglycerol
DGMG	Digalactosyl monoacylglycerol
DGTA	Diacylglyceryl-hydroxymethyltrimethyl-β-alanine
DGTS	Diacylglyceryl-3-O-4-*N,N,N*-trimethyl-homoserine
HILIC	Hydrophilic interaction liquid chromatography
IMTA	Integrated multitrophic aquaculture
LPC	Lyso-phosphatidylcholine
LPE	Lyso-phosphatidylethanolamine
LPG	Lyso-phosphatidylglycerol
LPI	Lyso-phosphatidylinositol
m/z	Mass to charge ratio
MGDG	Monogalactosyl diacylglycerol
MGMG	Monogalactosyl monoacylglycerol
MGTA	Monoacylglyceryl-hydroxy methyl-trimethyl-β-alanine
MGTS	Monocylglyceryl-3-O-4-(*N,N,N*-trimethyl) homoserine
MS	Mass spectrometry
MS/MS	Tandem mass spectrometry
MUFA	Monounsaturated fatty acid
PC	Phosphatidylcholine
PE	Phosphatidylethanolamine
PG	Phosphatidylglycerol
PI	Phosphatidylinositol
PL	Phospholipid
PUFA	Polyunsaturated fatty acid
RT	Retention time
SFA	Saturated fatty acid
SQDG	Sulfoquinovosyl diacylglycerol
SQMG	Sulfoquinovosyl monoacylglycerol
