# Supplementary material for: Lipidomic Signatures Reveal Seasonal Shifts on the Relative Abundance of High-Valued Lipids from the Brown Algae Fucus vesiculosus
Source: Mar Drugs. 2019 Jun 4;17(6):335. doi: 10.3390/md17060335 (PMC6627367; doi:10.3390/md17060335)
Supplement: Supplementary file 1 [file marinedrugs-17-00335-s001.pdf]

Supplementary information

Lipidomic signatures reveal seasonal shifts on the relative abundance of high-valued lipids from the brown algae *Fucus vesiculosus*

Elisabete da Costa [1,2], Pedro Domingues [1],Tânia Melo [1,2], Elisabete Coelho [3], Rui Pereira [4], Ricardo Calado [5], Maria H. Abreu [4], M. Rosário Domingues [1,2,*]

[1] Centro de Espetrometria de Massa, Departamento de Química & QOPNA& LAQV-REQUIMTE, Universidade de Aveiro, Campus Universitário de Santiago, 3810-193 Aveiro

[2] Departamento de Química & CESAM & ECOMARE, Universidade de Aveiro, Campus Universitário de Santiago, 3810-193 Aveiro, Portugal

[3] Departamento de Química & QOPNA & LAQV-REQUIMTE, Universidade de Aveiro, Campus Universitário de Santiago, 3810-193 Aveiro

[4] ALGAplus - Produção e comercialização de algas e seus derivados, Lda., 3830-196 Ílhavo, Portugal

[5] Departamento de Biologia & CESAM & ECOMARE, Universidade de Aveiro, Campus Universitário de Santiago, 3810-193 Aveiro, Portugal

* Correspondence: mrd@ua.pt

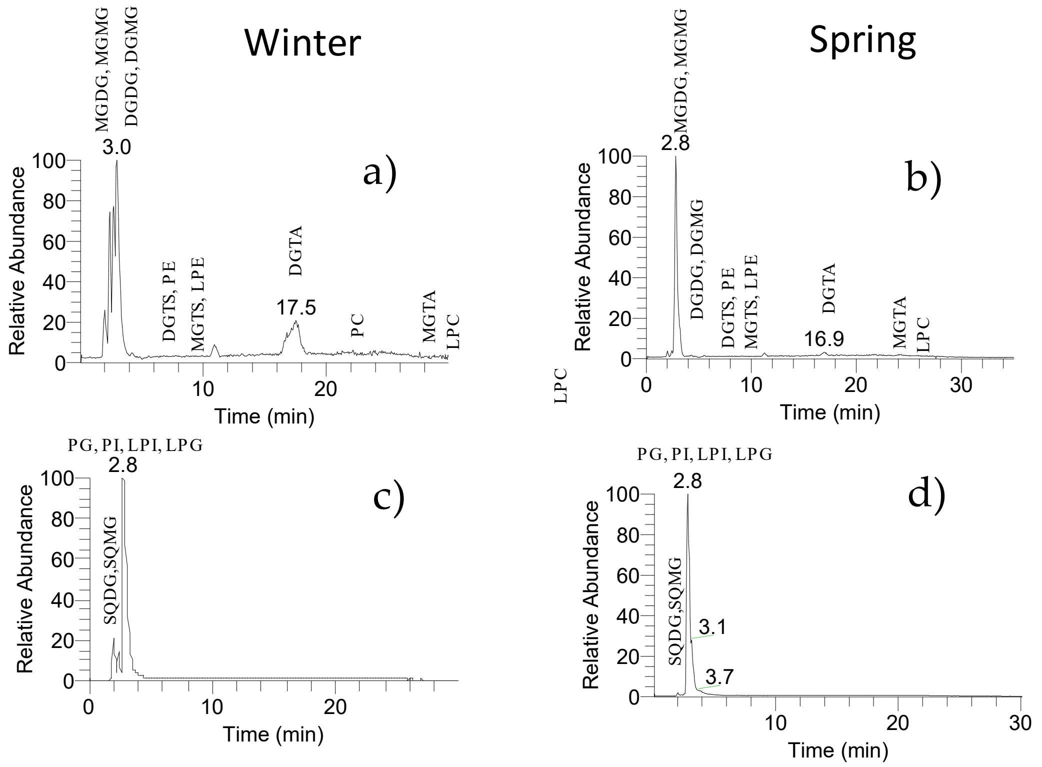

Figure S1. Total ion chromatograms (TIC) of lipid extracts from *Fucus vesiculosus* collected in winter and spring, respectively acquired on positive mode (a, b) and negative mode (c, d).

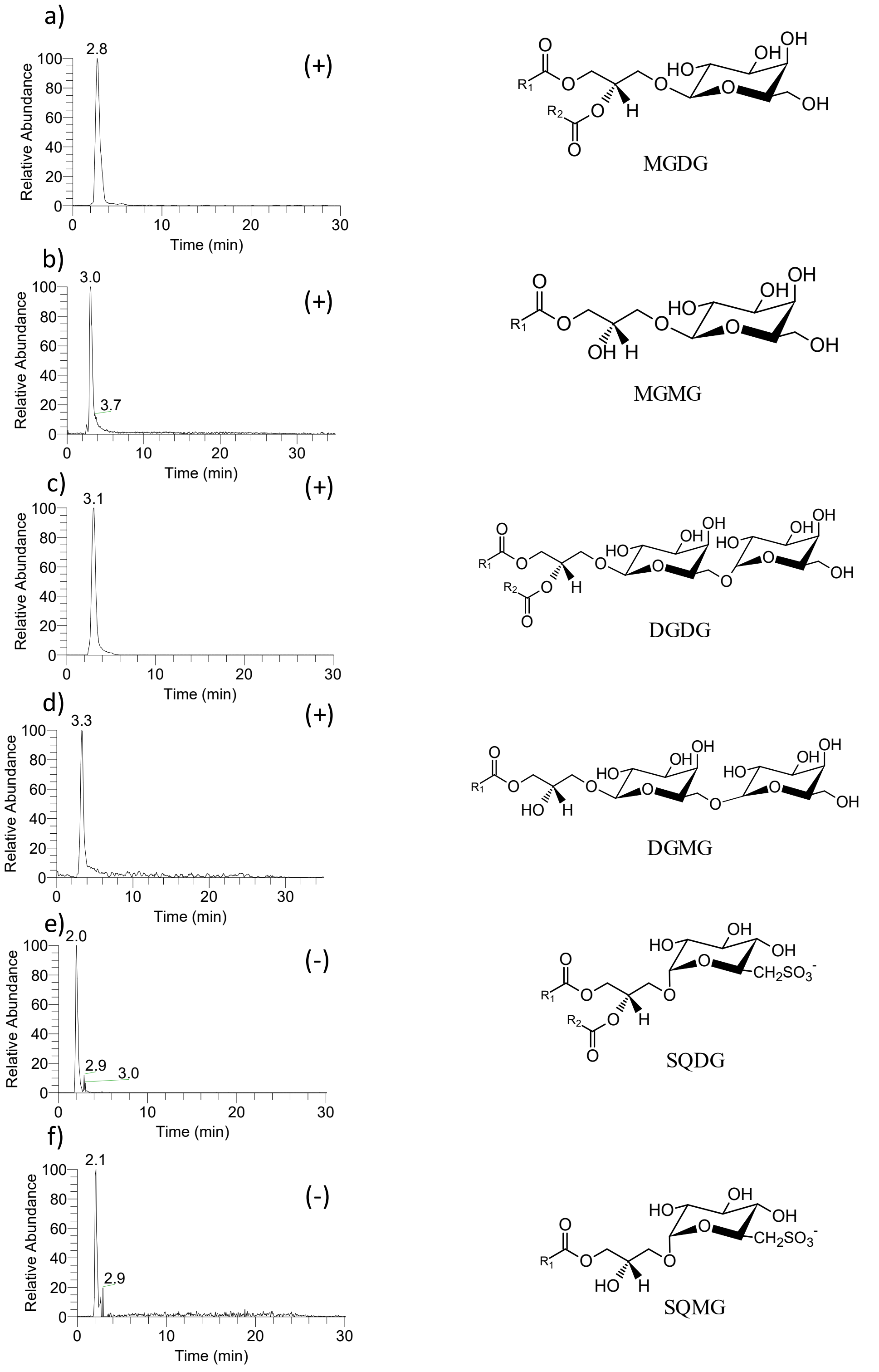

Figure S2. Reconstructed ion chromatograms (RIC) representative of the glycolipid classes. RIC of the $[M + NH_4]^+$ ions of the neutral glycolipids MGDG at *m/z* 816.5626 (a), MGMG at *m/z* 530.3329 (b), DGDG at *m/z* 978.6154 (c), DGMG at *m/z* 694.4014 (d), and of the $[M - H]^-$ ions of the acidic glycolipids SQDG at *m/z* 819.5292 (e) and SQMG at *m/z* 555.2839 (f).

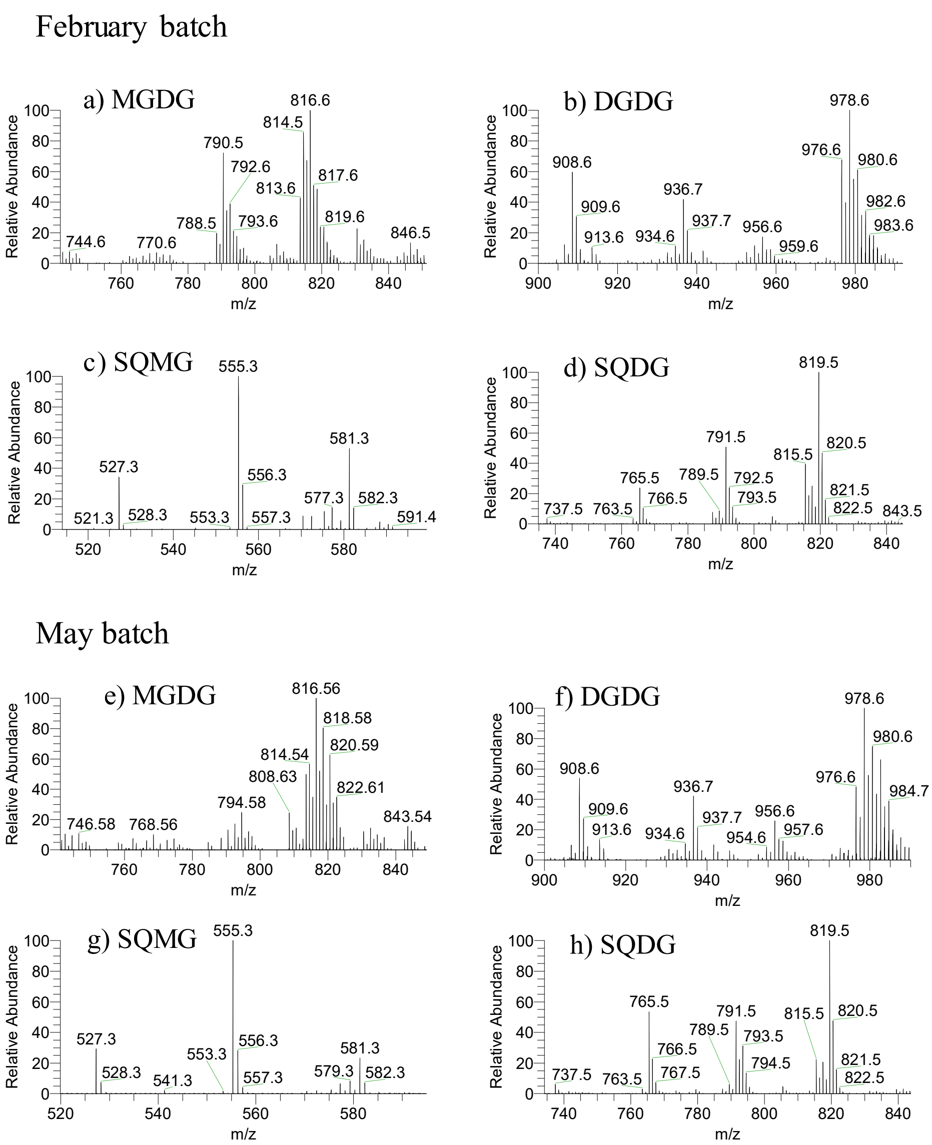

Figure S3. LC–MS spectra of glycolipids from *Fucus vesiculosus* collected in winter (February batch, a, b, c, d) and spring (May batch e, f, g, h). MGDG (a and e), DGDG (b and f) molecular species were identified as $[M + NH_4]^+$ ions. SQMG (c and g) and SQDG (d and h) were identified as $[M - H]^-$ ions. The accurate mass of *m/z* values with 4 decimal places are specified in table S1.

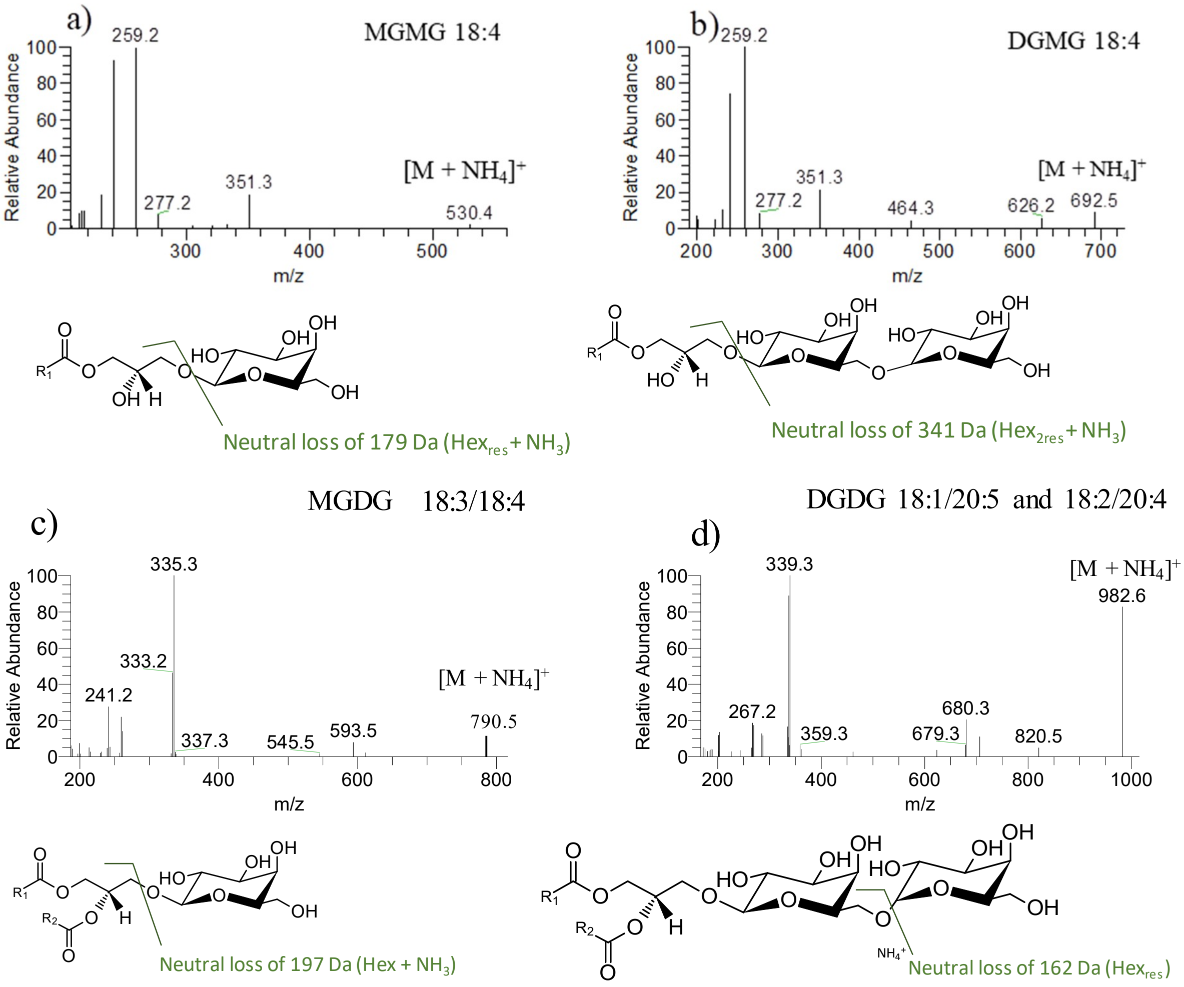

Figure S4. LC–MS/MS spectra of $[M + NH_4]^+$ ions of MGMG (18:4) at *m/z* 530.4 (a), DGMG (18:4) at *m/z* 692.5 (b), MGDG (18:3/18:4) at *m/z* 790.5 (c) and DGDG (18:1/20:5) - (18:2/20:4) at *m/z* 982.6 (d). Typical fragmentation of mono galactosyl glycolipids observed in the MS/MS spectra of the MGMG (a) is neutral loss of NH_3 plus loss of galactosyl residue (-179 Da) with formation of the ion at *m/z* 351.3, and in the case of MGDG (c) is the neutral loss of NH_3 plus loss of galactosyl unit (-197 Da) with formation of the ion at *m/z* 593.5. Typical fragmentation of digalactosyl glycolipids observed in the MS/MS spectra of the DGMG (b) is the neutral loss of NH_3 plus digalactosyl residue that correspond to the loss of 341 Da $[M + H - 341]^+$, herein seen as ion at *m/z* 351.3. In the case of DGDG (d) the typical neutral loss arise from (-162 Da) of the galactosyl residue herein seen as ion $[M + NH_4 – 162]^+$ at *m/z* 820.5 seen as ion. These fragmentation pathways allow to identify the polar head group. The typical product ions that allow to identify the fatty acyl composition are the acylium ion $[RCO]^+$ observed in the MS/MS spectra of the lyso glycolipids MGMG and DGMG, and the product ions $[RCO + 74]^+$ observed in the MS/MS spectra of the glycolipids MGDG and DGDG. In the MS/MS spectra of MGMG and DGMG the product ions $[RCO]^+$ are seen at *m/z* 259.3 (18:4 FA). In MS/MS spectrum of MGDG (c) the product ion $[RCO + 74]^+$ is seen at *m/z* 333.2 (18:4 FA) and at *m/z* 335.3 (18:3 FA) while in the MS/MS spectrum of DGDG the product ion $[RCO + 74]^+$ is seen at *m/z* 339.2 (18:1 FA) and at *m/z* 359.3 (20:5 FA) (d) [1-6].

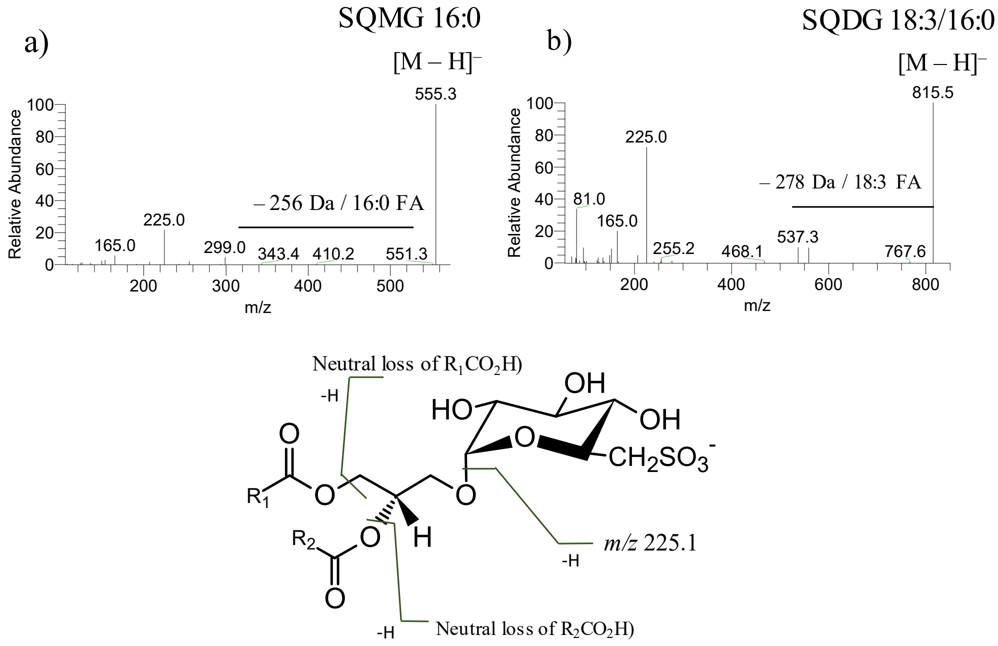

Figure S5. LC–MS/MS spectra of $[M - H]^-$ ions of SQMG (16:0) at *m/z* 555.3 (a), and SQDG (18:3/16:0) at *m/z* 815.5 (b). The diagnostic ions of both sulfolipid classes is the product ion at *m/z* 225.0, that correspond to polar head group sulfoquinovosyl anion $C_6H_9O_7S^-$ and the ion at *m/z* 81 assigned as SO_3^-. Product ions that allow to confirm fatty acyl composition are the product ion corresponding to neutral loss of fatty acyl group as free carboxylic (-RCOOH) identified at *m/z* 299.0 (– 256 Da, 16:0 FA) in the MS/MS spectrum of SQMG (a) and identified at *m/z* 537.3 (– 278 Da, 18:3 FA) and at 559.3 (– 256 Da, 16:0 FA) in the MS/MS spectra of SQDG (b). Typical abundant R_2COO^- product seen at *m/z* 255.2 corresponds to 16:0 FA [1-6].

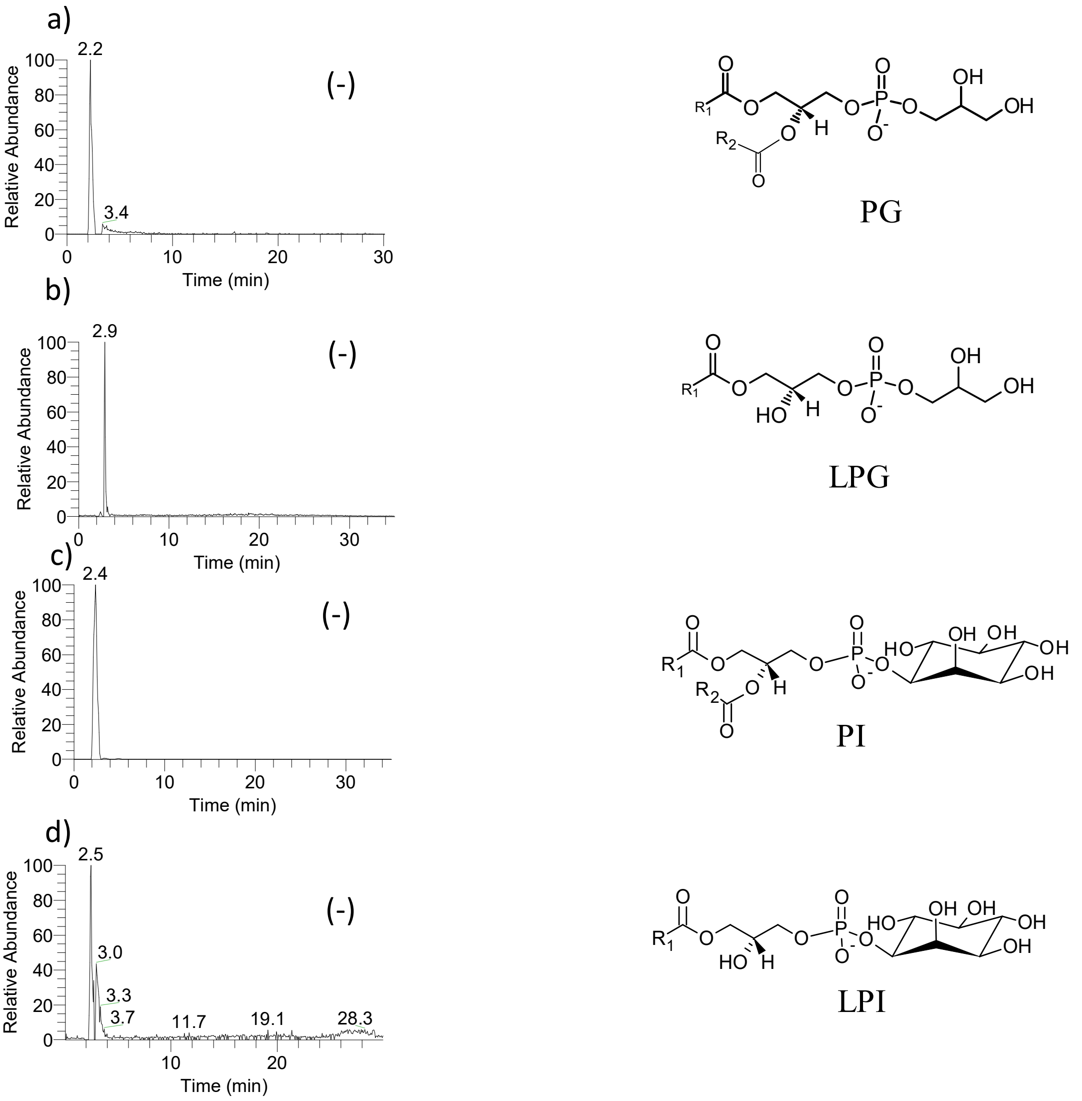

Figure S6. Reconstructed ion chromatograms (RIC) representative of the anionic phospholipid classes PG, LPG, PI and LPI classes: RIC of the [M – H]⁻ ions of the PG at *m/z* 741.4707 (a), LPG at *m/z* 481.2566 (b), PI at *m/z* 835.5337 (c) and LPI at *m/z* 597.3040 (d).

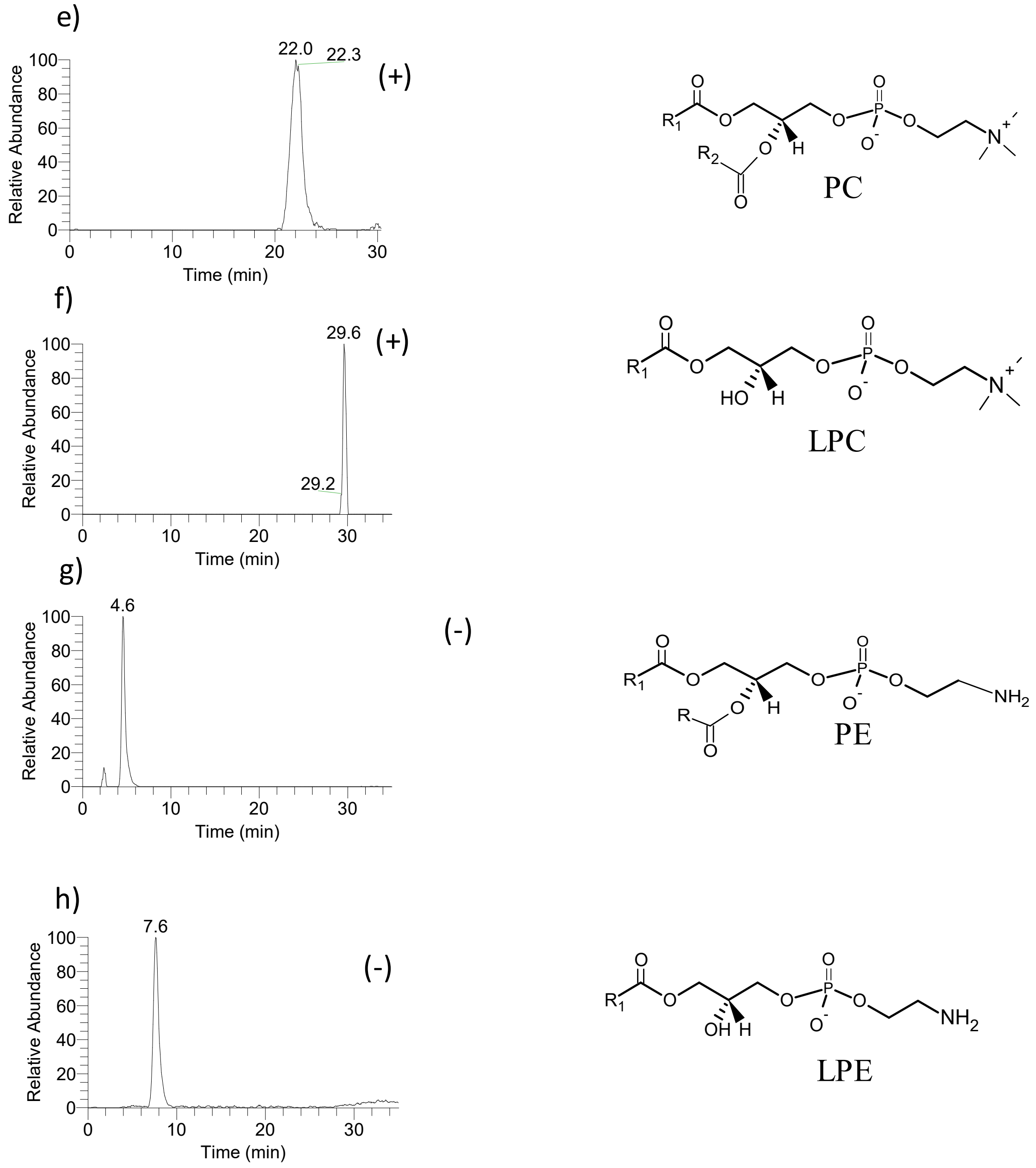

Figure S7. Reconstructed ion chromatograms (RIC) representative of the phospholipid classes PC, LPC, PE and LPE: RIC of the $[M + H]^+$ ions of PC at *m*/*z* 784.5856 (e), LPC at *m*/*z* 496.3403 (f) and of the $[M – H]^–$ ions of the PE at *m*/*z* 786.5074 (g) and LPE at *m*/*z* 500.2777 (h).

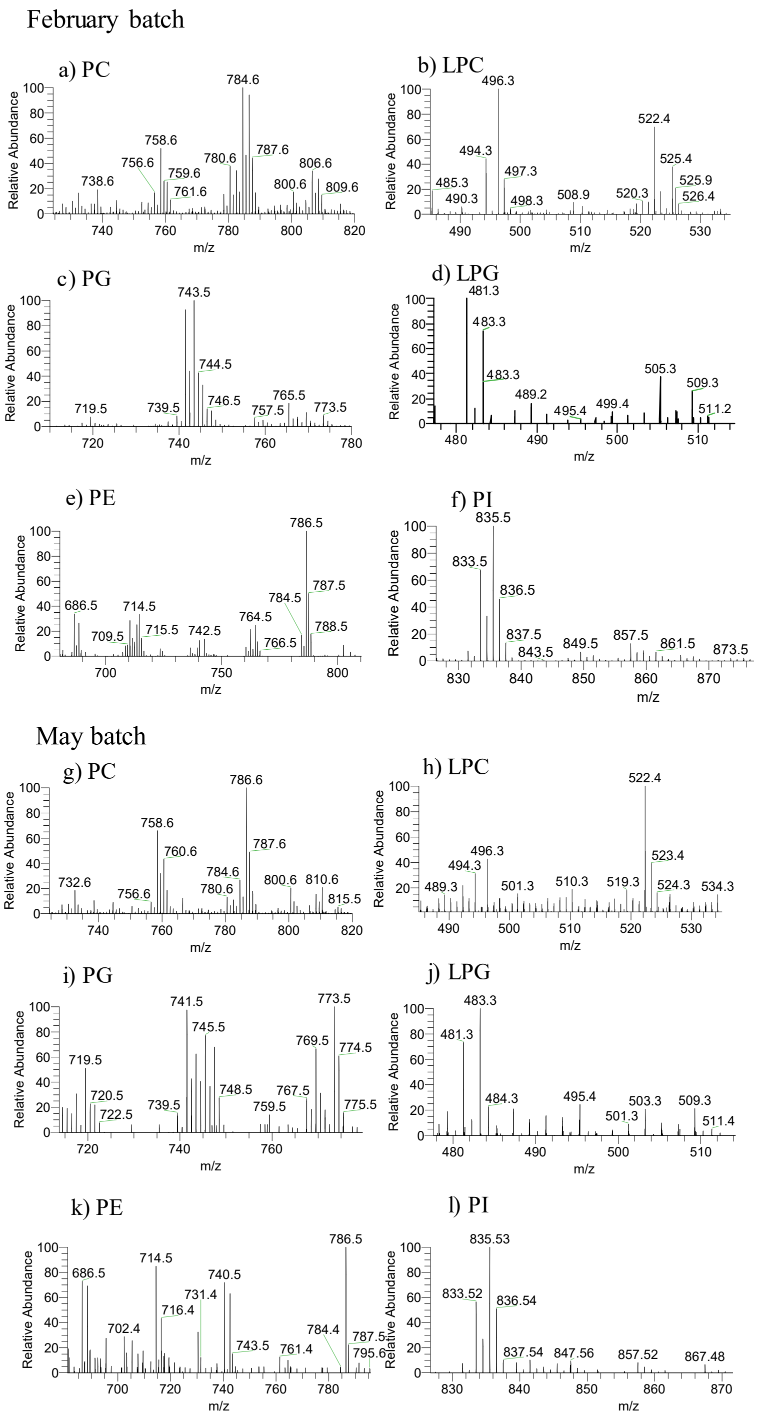

Figure S8. LC–MS spectra of phospholipids from *Fucus vesiculosus* collected in winter (February batch a, b, c, d, e, f) and spring (May batch g, h, I, j, k, l). PC (a and g), LPC (b and h) were identified as $[M + H]^+$ ions; PG (c and i), LPG (d and j), PE (e and k), and PI (f and l) were identified as $[M - H]^-$ ions. The accurate mass of *m/z* values with 4 decimal places are specified in table S1.

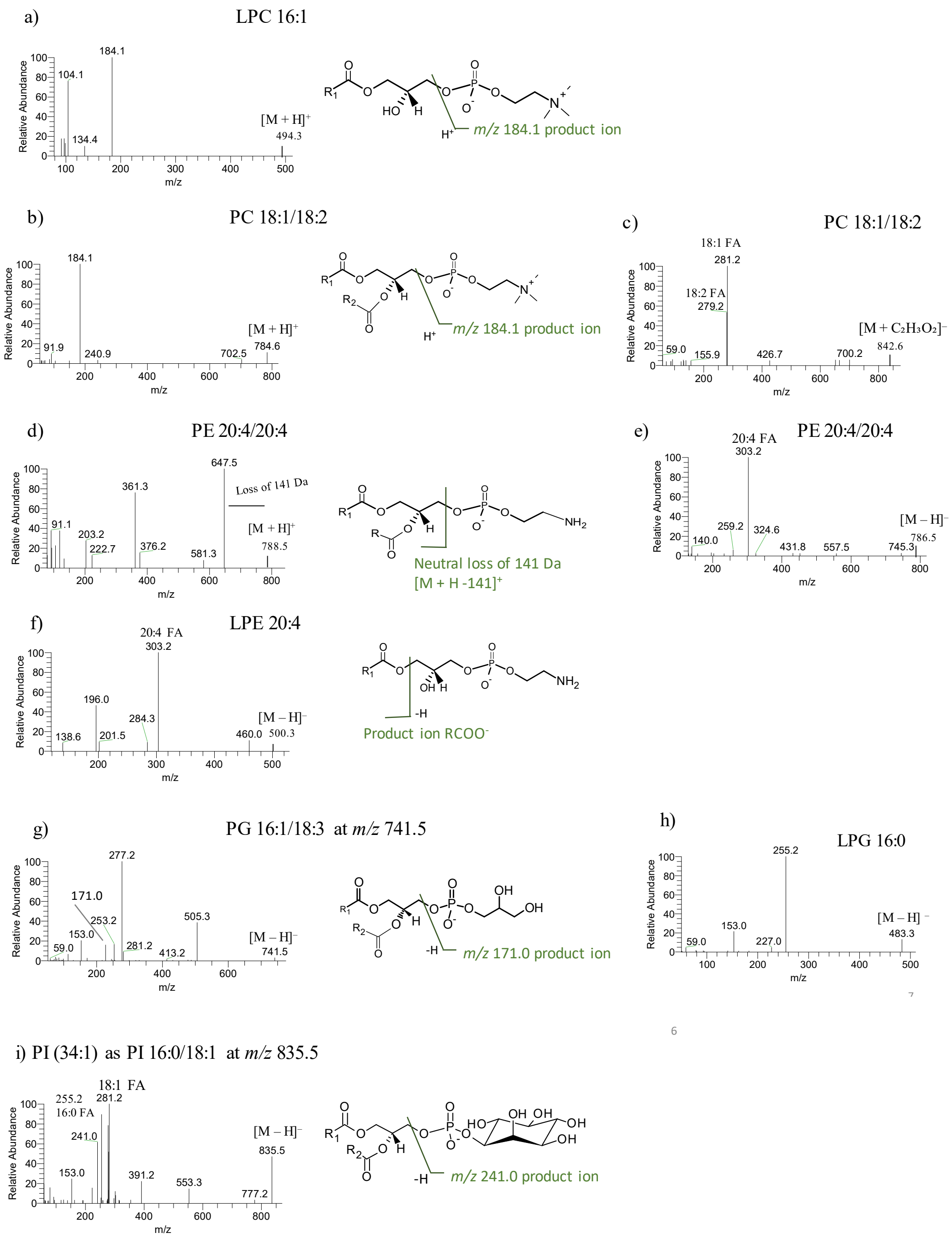

Figure S9. MS/MS spectra of LPC 16:1 at *m/z* 494.3 as $[M + H]^+$ (a), PC 18:1/18:2 as $[M + H]^+$ at *m/z* 784.6 (b)and as $[M + CH_3OO]^-$ at *m/z* 842.6 (c), PE 20:4/20:4 at *m/z* 788.5 as $[M + H]^+$ (d) and at *m/z* 786.5 as $[M – H]^-$ (e), LPE 20:4 at *m/z* 500.3 as $[M – H]^-$ (f), PG 16:1/18:3 at m/z 741.5 as $[M – H]^-$ (g), LPG 16:0 at *m/z* 483.3 as $[M – H]^-$ (h), and PI 16:0/18:1 at *m/z* 835.5 as $[M – H]^-$ (i). (a) and (b) LPC and PC class are characterized by the abundant product ion formed in positive mode at *m/z* 184.1 that is PC diagnostic ion phosphocholine $C_5H_{15}NO_4P^+$. (c) Fatty acyl groups are assigned by abundant carboxylate anions R_1COO^- and R_2COO^- observed in the MS/MS spectra of $[M + CH_3OO]^-$ as seen herein at *m/z* 279.2 (18:2 FA) and 281.2 (18:1 FA). (e)

and (f) PE and LPE classes fatty acyl composition is identified in the MS/MS spectra of the [M – H]$^-$ ion by the presence of abundant carboxylate anions R_1COO^- seen in this case at *m*/*z* 303.2 (20:4 FA). In the positive mode (d) the PE class is confirmed by the abundant product ion formed by neutral loss of 141 Da due to loss of polar head (*m*/*z* 647.5). (g) PG fatty acyl composition was assigned by R_1COO^- and R_2COO^- (*m*/*z* 253.2 – 16:1 FA and 277.2 – 18:3 FA) and ions corresponding to neutral loss of ketene [M – H – RCHC=C=O]$^-$ at *m*/*z* 505 (-236 Da, ketene form of 16:1 FA). Additionally, ion at *m*/*z* 413.2 arise to loss of fatty acyl group as free carboxylic acid (-278 Da – 18:3 FA) along with polar head group (–135 Da, $C_4H_5O_4P^-$) as well as *m*/*z* ion 171 (glycerol phosphate). (h) LPG fatty acyl composition was assigned by abundant product ion $RCOO^-$ (*m*/*z* 255.2). (i) PI class was characterized by diagnostic product ion at *m*/*z* 241.0 identified as cyclic anion of inositol phosphate. Abundant R_1COO^- and R_2COO^- (16:0 FA as *m*/*z* 255.2 and 18:1 FA as 281.2 ions) and ions corresponding to neutral loss of each fatty acyl group as free carboxylic acid [M – H – RCOOH]$^-$ at *m*/*z* 553.3 (neutral loss of 282 Da represents18:1 FA9 are also seen [1-6].

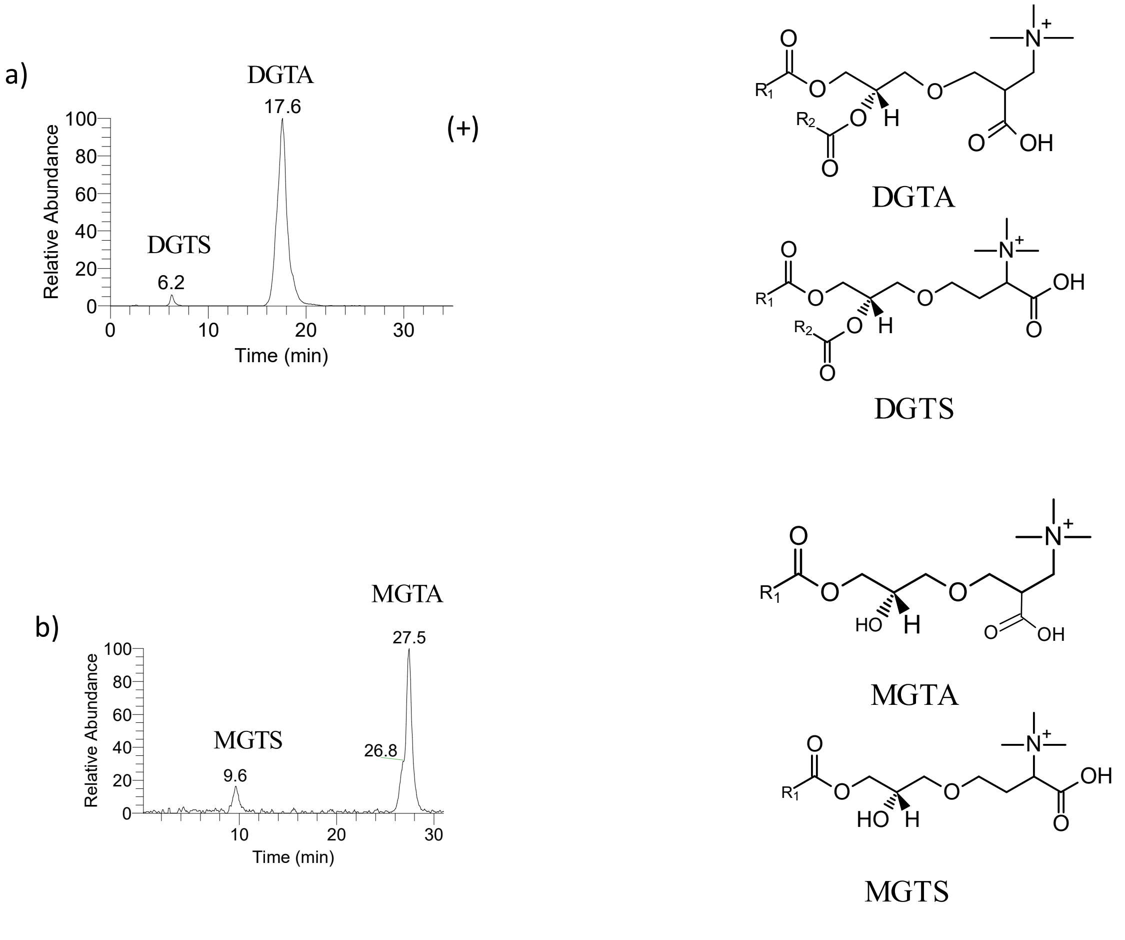

Figure S10. Reconstructed ion chromatograms representative of the betaine lipids: RIC of the [M + H]$^+$ ions of the DGTS and DGTA at *m*/*z* 708.5778 (a) and of the MGTS and MGTA at *m*/*z* 498.3795 (b).

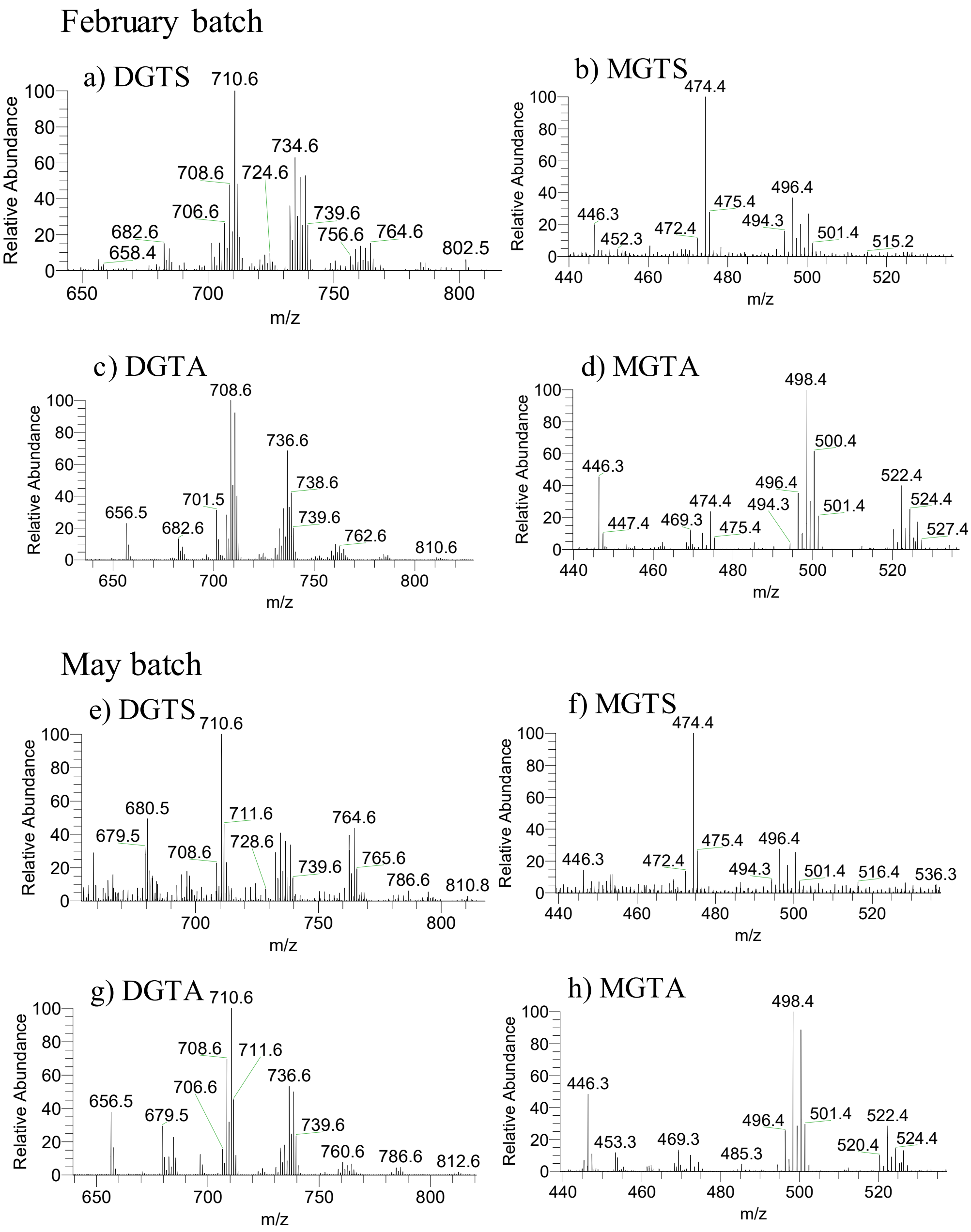

Figure S11. LC–MS spectra of betaine lipids from *Fucus vesiculosus* collected in winter (February batch a, b, c, d) and spring (May batch e, f, g, h). DGTS (a and e), MGTS (b and f), DGTA (c and g), MGTA (e and h) were identified as $[M + H]^+$ ions. The accurate mass of *m*/*z* values with 4 decimal places are specified in table S1.

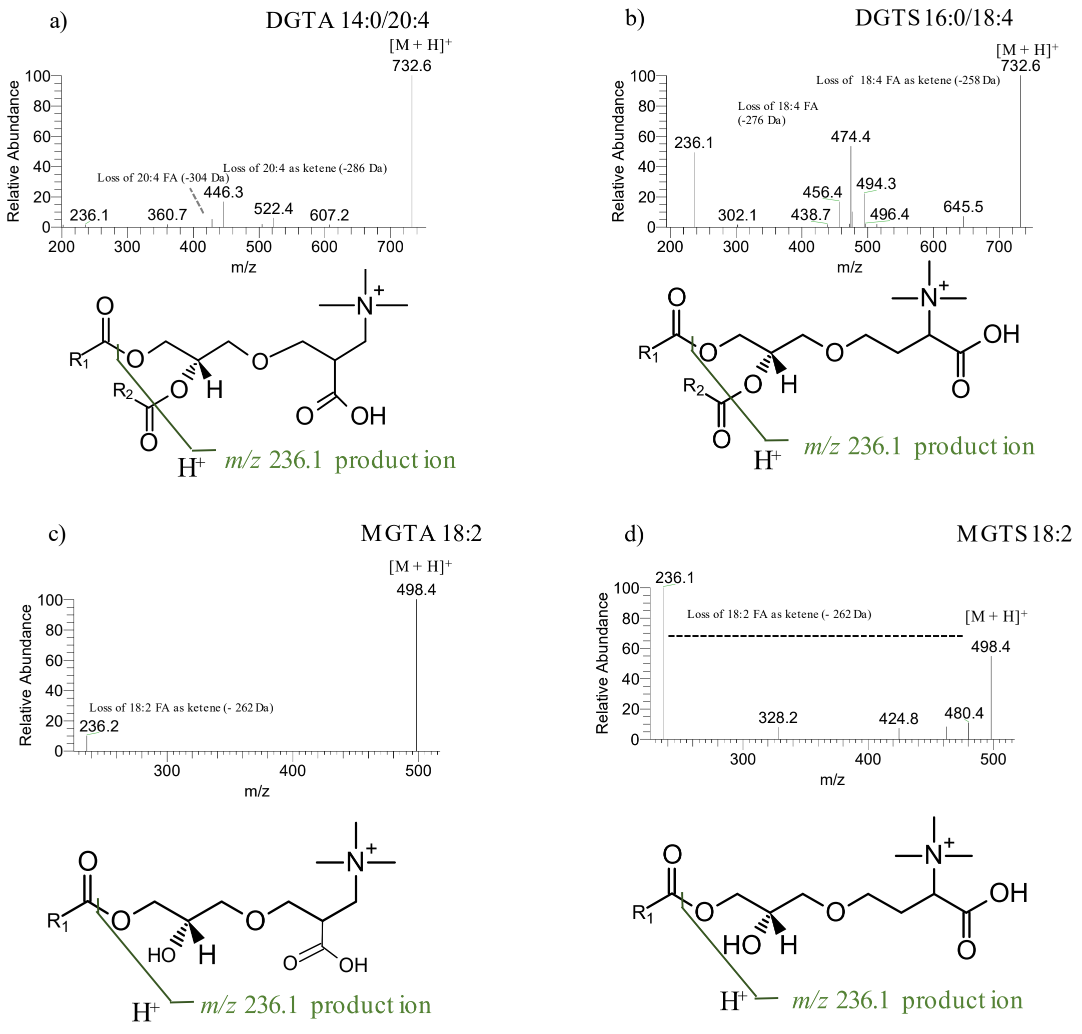

Figure S12. MS/MS spectra of DGTA (34:4) at *m/z* 732.6 (a), DGTS (34:4) at *m/z* 732.6 (b), MGTA (34:4) at (18:2) at *m/z* 498.46 (c) and MGTS (18:2) at *m/z* 498.46 (d). The product ions resultant from neutral loss of either $R_{1,2}COOH$ free carboxylic acids and ketene $R_{1,2}CH=C=O$ that are indicative of the respective fatty acyl composition of the molecular species. The characteristic ion at *m/z* 236.150 arises from polar head is the diagnostic ion of these classes [2-6].

Annotations of tandem mass spectrometry were based on literature:

1. Murphy, R.C. Tandem mass spectrometry of lipids. New developments in Mass spectrometry No.4. ISBN 978-11-84973-827-9. Royal Society of Chemistry, **2015**.
2. Melo, T.; Alves, E.; Azevedo, V.; Martins, A. S.; Neves, B.; Domingues, P.; Calado, R.; Abreu, M. H.; Domingues, M. R. Lipidomics as a new approach for the bioprospecting of marine macroalgae — Unraveling the polar lipid and fatty acid composition of *Chondrus crispus*. *Algal Res.* **2015**, *8*, 181–191.
3. da Costa, E.; Melo, T.; Moreira, A. S. P. A. S. P.; Alves, E.; Domingues, P.; Calado, R.; Abreu, M. H. M. H.; Domingues, M. R. Decoding bioactive polar lipid profile of the macroalgae *Codium tomentosum* from a sustainable IMTA system using a lipidomic approach. *Algal Res. Submitt.* **2015**, *12*, 388–397.
4. Da Costa, E.; Melo, T.; Moreira, A. S. P. A.; Bernardo, C.; Helguero, L.; Ferreira, I.; Cruz, M. T. M.; Rego,

A. M. A.; Domingues, P.; Calado, R.; Abreu, M. M. H.; Domingues, M. Valorization of lipids from *Gracilaria* sp. through lipidomics and decoding of antiproliferative and anti-inflammatory Activity. *Mar. Drugs* **2017**, *15*, 62.

5. da Costa, E.; Azevedo, V.; Melo, T.; Rego, A. M.; Evtuguin, D. V.; Domingues, P.; Calado, R.; Pereira, R.; Abreu, M. H.; Domingues, M. R. High-resolution lipidomics of the early life stages of the red seaweed *Porphyra dioica*. *Molecules* **2018**, *23*.
6. Lopes, D.; Moreira, A. S. P.; Rey, F.; da Costa, E.; Melo, T.; Maciel, E.; Rego, A.; Abreu, M. H.; Domingues, P.; Calado, R.; Lillebø, A. I.; Rosário Domingues, M. Lipidomic signature of the green macroalgae *Ulva rigida* farmed in a sustainable integrated multi-trophic aquaculture. *J. Appl. Phycol.* **2018**, 2, 1369-1381

Table S1. Molecular species observed by HILIC–MS, with the assignment of the total fatty acyl composition of each lipid molecular species. Analyses were determined by mass accuracy (error ≤ 5 ppm) using the MZ mine 2 and Xcalibur software, exact mass calculator http: //www.sisweb.com/referenc/tools/ exactmass.htm, and lipid maps tools http://www.lipidmaps.org/tools/ and home database for glycolipids and betaine lipids (Mass spectrometry Center of Aveiro)

Formula	Lipid species (C:N)	Calculated *m/z*	Observed *m/z*	Delta ppm
C41H80NO10	MGDG (32:1)	746.5777	746.5765	-1.6073
C43H78NO10	MGDG (34:4)	768.5620	768.5621	0.1301
C43H80NO10	MGDG (34:3)	770.5782	770.5764	-2.3671
C43H82NO10	MGDG (34:2)	772.5933	772.5935	0.2589
C43H84NO10	MGDG (34:1)	774.6090	774.6074	-2.0656
C45H74NO10	MGDG (36:8)	788.5313	788.5307	-0.7229
C45H76NO10	MGDG (36:7)	790.5469	790.5459	-1.2649
C45H78NO10	MGDG (36:6)	792.5625	792.5614	-1.3879
C45H80NO10	MGDG (36:5)	794.5782	794.5772	-1.2585
C45H82NO10	MGDG (36:4)	796.5933	796.5929	-0.5021
C47H76NO10	MGDG (38:9)	814.5469	814.5449	-2.4848
C47H78NO10	MGDG (38:8)	816.5626	816.5608	-2.1676
C47H80NO10	MGDG (38:7)	818.5782	818.5766	-1.9839
C47H82NO10	MGDG (38:6)	820.5939	820.5926	-1.5842
C47H84NO10	MGDG (38:5)	822.6095	822.6073	-2.6987
C47H86NO10	MGDG (38:4)	824.6251	824.6235	-1.9403
C49H80NO10	MGDG (40:9)	842.5782	842.5751	-3.6978
C49H82NO10	MGDG (40:8)	844.5939	844.5940	0.0983
C27H48NO9	MGMG (18:4)	530.3329	530.3327	-0.3941
C27H50NO9	MGMG (18:3)	532.3486	532.3488	0.4527
C27H52NO9	MGMG (18:2)	534.3642	534.3646	0.7317
C27H54NO9	MGMG (18:1)	536.3799	536.3795	-0.6693
C47H88O15N	DGDG (32:2)	906.6154	906.6148	-0.6607
C47H90O15N	DGDG (32:1)	908.6310	908.6305	-0.6042
C49H88O15N	DGDG (34:4)	930.6154	930.6135	-2.0406
C49H90O15N	DGDG (34:3)	932.6310	932.6305	-0.5887
C49H92O15N	DGDG (34:2)	934.6467	934.6461	-0.6409
C49H94O15N	DGDG (34:1)	936.6623	936.6618	-0.5861
C49H96O15N	DGDG (34:0)	938.6780	938.6780	0.0011
C51H86O15N	DGDG (36:7)	952.5997	952.5992	-0.5763

C51H88O15N	DGDG (36:6)	954.6154	954.6148	-0.6275
C51H90O15N	DGDG (36:5)	956.6310	956.6305	-0.5739
C51H92O15N	DGDG (36:4)	958.6467	958.6461	-0.6248
C51H94O15N	DGDG (36:3)	960.6623	960.6623	-0.0510
C51H96O15N	DGDG (36:2)	962.6780	962.6780	0.0010
C51H98O15N	DGDG (36:1)	964.6936	964.6936	-0.0508
C53H86O15N	DGDG (38:9)	976.5997	976.5997	-0.0502
C53H88O15N	DGDG (38:8)	978.6154	978.6144	-1.0208
C53H90O15N	DGDG (38:7)	980.6310	980.6289	-2.1914
C53H92O15N	DGDG (38:6)	982.6467	982.6448	-1.9325
C53H94O15N	DGDG (38:5)	984.6623	984.6594	-2.9949
C53H96O15N	DGDG (38:4)	986.6780	986.6767	-1.3165
C31H62NO14	DGMG (16:0)	672.4170	672.4167	-0.4908
C33H58NO14	DGMG (18:4)	692.3857	692.3856	-0.1935
C33H60NO14	DGMG (18:3)	694.4014	694.4011	-0.4090
C33H62NO14	DGMG (18:2)	696.4170	696.4159	-1.6283
C33H64NO14	DGMG (18:1)	698.4327	698.4321	-0.8362
C37H69O12S	SQDG (28:0)	737.4510	737.4495	-2.0028
C39H71O12S	SQDG (30:1)	763.4666	763.4648	-2.3930
C39H73O12S	SQDG (30:0)	765.4823	765.4804	-2.4520
C41H71O12S	SQDG (32:3)	787.4666	787.4646	-2.5741
C41H73O12S	SQDG (32:2)	789.4823	789.4804	-2.3775
C41H75O12S	SQDG (32:1)	791.4979	791.4958	-2.6873
C41H77O12S	SQDG (32:0)	793.5136	793.5099	-4.6326
C43H73O12S	SQDG (34:4)	813.4823	813.4803	-2.4303
C43H75O12S	SQDG (34:3)	815.4979	815.4958	-2.6082
C43H77O12S	SQDG (34:2)	817.5136	817.5107	-3.5180
C43H79O12S	SQDG (34:1)	819.5292	819.5269	-2.8394
C45H75O12S	SQDG (36:5)	839.4979	839.4977	-0.2704
C45H77O12S	SQDG (36:4)	841.5136	841.5106	-3.5365
C23H43O11S	SQMG (14:0)	527.2526	527.2515	-2.1090
C25H45O11S	SQMG (16:1)	553.2683	553.2664	-3.3655
C25H47O11S	SQMG (16:0)	555.2839	555.2829	-1.8225
C27H45O11S	SQMG (18:3)	577.2683	577.2673	-1.6665
C27H47O11S	SQMG (18:2)	579.2839	579.2832	-1.2291
C27H49O11S	SQMG (18:1)	581.2996	581.2984	-1.9972
C38H71NO8P	PC (32:2)	730.5350	730.5380	-4.0878
C42H79NO8P	PC (34:3)	756.5536	756.5550	-1.8591

C42H81NO8P	PC (34:2)	758.5700	758.5689	-1.4264
C42H83NO8P	PC (34:1)	760.5856	760.5835	-2.8031
C44H79NO8P	PC (36:5)	780.5543	780.5530	-1.7052
C44H83NO8P	PC (36:3)	784.5856	784.5850	-0.8055
C44H85NO8P	PC (36:2)	786.6013	786.6005	-0.9942
C46H79NO8P	PC (38:7)	804.5533	804.5545	-1.4920
C46H81NO8P	PC (38:6)	806.5700	806.5683	-2.0854
C46H83NO8P	PC (38:5)	808.5856	808.5831	-3.1314
C24H51NO7P	LPC (16:0)	496.3403	496.3392	-2.2485
C24H49NO7P	LPC (16:1)	494.3247	494.3241	-1.1470
C26H53NO7P	LPC (18:1)	522.3560	522.3556	-0.7026
C38H70O10P	PG (32:2)	717.4707	717.471	0.4181
C38H72O10P	PG (32:1)	719.4863	719.4868	0.6949
C38H74O10P	PG (32:0)	721.5020	721.5033	1.8018
C40H70O10P	PG (34:4)	741.4707	741.4713	0.8092
C40H72O10P	PG (34:3)	743.4863	743.4862	-0.1345
C40H74O10P	PG (34:2)	745.5020	745.5018	-0.2683
C40H76O10P	PG (34:1)	747.5176	747.5183	0.9364
C40H78O10P	PG (34:0)	749.5333	749.5324	-1.2007
C42H74O10P	PG (36:4)	769.5020	769.4987	-4.2885
C42H76O10P	PG (36:3)	771.5176	771.5160	-2.0738
C42H78O10P	PG (36:2)	773.5333	773.5321	-1.5513
C22H42O9P	LPG (16:1)	481.2566	481.2569	0.5236
C22H44O9P	LPG (16:0)	483.2723	483.2733	2.0734
C24H46O9P	LPG (18:1)	509.2879	509.2887	1.4766
C43H76O13P	PI (34:3)	831.5024	831.4984	-4.8106
C43H78O13P	PI (34:2)	833.5180	833.5175	-0.5999
C43H80O13P	PI (34:1)	835.5337	835.5339	0.2394
C43H82O13P	PI (34:0)	837.5493	837.5491	-0.2388
C45H78O13P	PI (36:4)	857.5180	857.5196	1.8659
C45H80O13P	PI (36:3)	859.5337	859.5352	1.7451
C45H82O13P	PI (36:2)	861.5493	861.5505	1.3928
C45H84O13P	PI (36:1)	863.5650	863.5674	2.7792
C27H50O12P	LPI (18:1)	597.3040	597.306	3.3601
C37H69O8NP	PE (32:2)	686.4761	686.4758	-0.4108
C39H73NO8P	PE (34:2)	714.5074	714.5067	-0.9545
C39H75NO8P	PE (34:1)	716.5230	716.5227	-0.4633

C43H73O8NP	PE (38:6)	762.51	762.5073	0.3934
C43H75NO8P	PE (38:5)	764.5230	764.5232	0.2616
C45H71O8NP	PE (40:9)	784.4917	784.4918	0.0867
C45H73O8NP	PE (40:8)	786.5074	786.5076	0.2772
C25H41NO7P	LPE (20:5)	498.2621	498.2613	-1.5394
C25H43NO7P	LPE (20:4)	500.2777	500.2779	0.3658
C38H74O7N	DGTS (28:0)	656.5465	656.5468	0.4128
C40H76O7N	DGTS (30:1)	682.5622	682.5628	0.9098
C40H78O7N	DGTS (30:0)	684.5778	684.5787	1.2723
C42H74O7N	DGTS (32:4)	704.5465	704.5458	-1.0347
C42H76O7N	DGTS (32:3)	706.5622	706.5617	-0.6779
C42H78O7N	DGTS (32:2)	708.5778	708.5778	-0.0409
C42H80O7N	DGTS (32:1)	710.5935	710.5933	-0.2519
C44H78O7N	DGTS (34:4)	732.5778	732.5774	-0.5856
C44H80O7N	DGTS (34:3)	734.5935	734.5932	-0.3798
C44H82O7N	DGTS (34:2)	736.6091	736.6083	-1.1241
C44H84O7N	DGTS (34:1)	738.6248	738.624	-1.0547
C46H84O7N	DGTS (36:3)	762.6248	762.6248	0.0275
C46H86O7N	DGTS (36:2)	764.6404	764.6405	0.0929
C24H48O6N	MGTS 14:0	446.34816	446.347	-2.6078
C26H50O6N	MGTS 16:1	472.36381	472.3632	-1.2998
C26H52O6N	MGTS 16:0	474.3795	474.3792	-0.5565
C28H48O6N	MGTS 18:4	494.3482	494.3477	-0.9386
C28H50O6N	MGTS 18:3	496.3638	496.3634	-0.8341
C28H52O6N	MGTS 18:2	498.3795	498.3796	0.2729
C28H54O6N	MGTS 18:1	500.3951	500.3943	-1.6267
C38H74O7N	DGTA (28:0)	656.5465	656.547	0.7174
C40H74O7N	DGTA (30:2)	680.5465	680.5433	-4.702
C40H76O7N	DGTA (30:1)	682.5622	682.5623	0.1773
C40H78O7N	DGTA (30:0)	684.5778	684.5782	0.5419
C42H74O7N	DGTA (32:4)	704.5465	704.5464	-0.1831
C42H76O7N	DGTA (32:3)	706.5622	706.5625	0.4543
C42H78O7N	DGTA (32:2)	708.5778	708.5778	-0.0409
C42H80O7N	DGTA (32:1)	710.5935	710.5933	-0.2519
C44H78O7N	DGTA (34:4)	732.5778	732.5776	-0.3126
C44H80O7N	DGTA (34:3)	734.5935	734.5935	0.0286
C44H82O7N	DGTA (34:2)	736.6091	736.6091	-0.0380

C44H84O7N	DGTA (34:1)	738.6248	738.6241	-0.9193
C46H80O7N	DGTA (36:5)	758.5935	758.5925	-1.3182
C46H82O7N	DGTA (36:4)	760.6091	760.6085	-0.8257
C46H84O7N	DGTA (36:3)	762.6248	762.6246	-0.2347
C46H84O7N	DGTA (36:2)	764.6404	764.6406	0.2616
C48H82O7N	DGTA (38:6)	784.6091	784.6084	-0.8922
C48H84O7N	DGTA (38:5)	786.6248	786.6235	-1.6526
C24H48O6N	MGTA 14:0	446.3482	446.3464	-3.9521
C26H50O6N	MGTA 16:1	472.3638	472.3618	-4.2637
C26H52O6N	MGTA 16:0	474.3795	474.3777	-3.7185
C28H48O6N	MGTA 18:4	494.3482	494.3463	-3.7706
C28H50O6N	MGTA 18:3	496.3638	496.3623	-3.0502
C28H52O6N	MGTA 18:2	498.3795	498.3778	-3.3388
C28H54O6N	MGTA 18:1	500.3951	500.3941	-2.0264
C30H50O6N	MGTA 20:5	520.3638	520.3619	-3.6513
C30H52O6N	MGTA 20:4	522.3795	522.3779	-2.9940
C30H54O6N	MGTA 20:3	524.3951	524.3958	1.3082

Table S2. Mann–Whitney *U* nonparametric test for univariate analysis of polar lipid transformed data set.

	F.value	p.value	FDR
MGDGC321	0	0.002165	0.007322
MGDGC344	6	0.064935	0.076087
MGDGC343	5	0.041126	0.053162
MGDGC342	21	0.699134	0.712579
MGDGC341	4	0.025974	0.034999
MGDGC368	36	0.002165	0.007322
MGDGC367	36	0.004922	0.007641
MGDGC366	30	0.064935	0.076087
MGDGC365	15.5	0.747921	0.757449
MGDGC364	7	0.092125	0.105381
MGDGC389	34	0.008658	0.012866
MGDGC388	30	0.064935	0.076087
MGDGC387	0	0.004922	0.007641
MGDGC386	0	0.004998	0.007641
MGDGC385	0	0.002165	0.007322
MGDGC384	7	0.093074	0.105705
MGDGC409	8	0.132035	0.147842
MGDGC408	0	0.002165	0.007322

DGDGC321	33	0.020022	0.027926
DGDGC322	36	0.004998	0.007641
DGDGC340	27.5	0.140194	0.15588
DGDGC341	5	0.044576	0.057157
DGDGC342	29	0.090412	0.10417
DGDGC343	34	0.011369	0.016585
DGDGC344	0	0.004922	0.007641
DGDGC361	28	0.086332	0.100195
DGDGC362	30	0.060856	0.076087
DGDGC363	14.5	0.618402	0.634361
DGDGC364	13.5	0.518159	0.537273
DGDGC365	7.5	0.108074	0.121871
DGDGC366	32	0.030348	0.040549
DGDGC367	36	0.003601	0.007641
DGDGC384	0	0.004337	0.007641
DGDGC385	0	0.004772	0.007641
DGDGC386	0	0.002165	0.007322
DGDGC387	10	0.228133	0.245089
DGDGC388	36	0.002165	0.007322
DGDGC389	36	0.004998	0.007641
PGC320	27	0.172733	0.186833
PGC321	0	0.002165	0.007322
PGC322	0	0.004998	0.007641
PGC340	4	0.025974	0.034999
PGC341	0	0.002165	0.007322
PGC342	13.5	0.520377	0.537273
PGC343	36	0.002165	0.007322
PGC344	36	0.002165	0.007322
PGC362	0	0.002165	0.007322
PGC363	20	0.818182	0.82336
PGC364	0	0.002165	0.007322
LPGC160	0	0.004998	0.007641
LPGC161	36	0.002165	0.007322
LPGC181	12	0.393939	0.41481
SQDGC280	0	0.004998	0.007641
SQDGC300	0	0.004922	0.007641
SQDGC301	35.5	0.0063	0.00954
SQDGC320	0	0.004772	0.007641
SQDGC321	36	0.004998	0.007641
SQDGC322	36	0.002165	0.007322
SQDGC323	36	0.002165	0.007322
SQDGC340	30	0.064935	0.076087

SQDGC341	36	0.004922	0.007641
SQDGC342	36	0.004922	0.007641
SQDGC343	36	0.004922	0.007641
SQDGC344	9	0.168947	0.185259
SQDGC364	2.5	0.015937	0.022424
SQDGC365	3.5	0.024722	0.03418
SQMGC140	3	0.015152	0.02151
SQMGC160	0	0.002165	0.007322
SQMGC161	3	0.015152	0.02151
SQMGC181	36	0.002165	0.007322
SQMGC182	1	0.004329	0.007641
SQMGC183	36	0.004922	0.007641
DGTAC280	0	0.002165	0.007322
DGTAC300	0	0.004922	0.007641
DGTAC301	36	0.004772	0.007641
DGTAC302	36	0.002165	0.007322
DGTAC321	0	0.004922	0.007641
DGTAC322	36	0.004998	0.007641
DGTAC323	36	0.002165	0.007322
DGTAC324	36	0.002165	0.007322
DGTAC341	0	0.004998	0.007641
DGTAC342	36	0.004624	0.007641
DGTAC343	36	0.004998	0.007641
DGTAC344	36	0.004998	0.007641
DGTAC362	1.5	0.010007	0.014732
DGTAC363	36	0.004998	0.007641
DGTAC364	36	0.002165	0.007322
DGTAC365	30	0.065081	0.076087
DGTAC385	5	0.041126	0.053162
DGTAC386	5	0.041126	0.053162
PIC340	36	0.002165	0.007322
PIC341	0	0.002165	0.007322
PIC342	0	0.002165	0.007322
PIC343	35	0.004329	0.007641
PIC361	36	0.004998	0.007641
PIC362	35	0.004329	0.007641
PIC363	36	0.002165	0.007322
PIC364	36	0.002165	0.007322
PEC322	0	0.002165	0.007322
PEC342	0	0.002165	0.007322
PEC341	9	0.158147	0.174621
PEC386	36	0.004772	0.007641

PEC385	35	0.004329	0.007641
PEC409	36	0.004772	0.007641
PEC408	36	0.004998	0.007641
LPEC204	0	0.002165	0.007322
LPEC205	36	0.002165	0.007322
DGMGC160	0	0.004998	0.007641
DGMGC181	0	0.002165	0.007322
DGMGC182	0	0.004847	0.007641
DGMGC183	36	0.004922	0.007641
DGMGC184	36	0.004998	0.007641
MGMGC181	2	0.012907	0.018656
MGMGC182	11	0.292834	0.312487
MGMGC183	36	0.004998	0.007641
MGMGC184	6	0.064935	0.076087
DGTSC280	30	0.064935	0.076087
DGTSC300	1	0.008127	0.012191
DGTSC301	0	0.002165	0.007322
DGTSC321	0	0.004998	0.007641
DGTSC322	36	0.002165	0.007322
DGTSC323	36	0.002165	0.007322
DGTSC324	36	0.002165	0.007322
DGTSC341	36	0.004772	0.007641
DGTSC342	36	0.002165	0.007322
DGTSC343	30	0.065081	0.076087
DGTSC344	23	0.484848	0.507177
DGTSC362	0	0.002165	0.007322
DGTSC363	5	0.041126	0.053162
MGTSC140	36	0.002165	0.007322
MGTSC160	36	0.002165	0.007322
MGTSC161	0	0.002165	0.007322
MGTSC181	35	0.004329	0.007641
MGTSC182	36	0.002165	0.007322
MGTSC183	36	0.002165	0.007322
MGTSC184	30	0.064935	0.076087
MGTAC140	18	1	1
MGTAC160	36	0.002165	0.007322
MGTAC161	9	0.171979	0.186833
MGTAC181	0	0.004922	0.007641
MGTAC182	0	0.004998	0.007641
MGTAC183	36	0.002165	0.007322
MGTAC184	0	0.004998	0.007641
MGTAC203	36	0.002165	0.007322

MGTAC204	36	0.004998	0.007641
MGTAC205	30	0.065081	0.076087
PCC322	36	0.004847	0.007641
PCC341	30	0.06461	0.076087
PCC342	0	0.004998	0.007641
PCC343	36	0.004847	0.007641
PCC362	0	0.004922	0.007641
PCC363	36	0.004698	0.007641
PCC365	36	0.004624	0.007641
PCC385	36	0.004772	0.007641
PCC386	36	0.004624	0.007641
PCC387	36	0.004847	0.007641
LPCC160	24	0.393939	0.41481
LPCC161	32	0.025974	0.034999
LPCC181	0	0.002165	0.007322
